# From Glycan Biology to Drug Candidates: An Integrated Sialylation Niche Index and AI-Guided Therapeutic Framework for Head and Neck Squamous Cell Carcinoma

**DOI:** 10.3390/biomedicines14092102

**Published:** 2026-09-17

**Authors:** Wei Gu, Chuan Liu, Jinglei Li, Jian Wang

**Affiliations:** 1 Department of Otolaryngology, Peking Union Medical College Hospital, Chinese Academy of Medical Sciences and Peking Union Medical College, Beijing 100730, China; ent_wei.gu@outlook.com (W.G.);; 2 CAMS Oxford Institute, Nuffield Department of Medicine, University of Oxford, Oxford OX3 7BN, UK

**Keywords:** sialylation, head and neck squamous cell carcinoma, prognostic model, machine learning, multi-omics, single-cell RNA sequencing, spatial transcriptomics, tumour microenvironment, drug discovery, HSPH1, ST6GALNAC1

## Abstract

**Background:** Head and neck squamous cell carcinoma (HNSCC) responds poorly (<20%) to immune checkpoint blockade. Since sialylation suppresses anti-tumour immunity through Siglec signalling independently of the PD-L1/PD-1 axis, we mapped its prognostic landscape in HNSCC by integrating prognostic modelling, molecular subtyping, and AI-guided drug discovery. **Methods:** Transcriptomic data from seven GEO cohorts and TCGA-HNSC (*n* = 501) were analysed with a 1204-gene sialylation compendium. The Sialylation Niche Index (SNI) was built in TCGA-HNSC from 117 machine-learning algorithm combinations (nominally 101), with survival-based feature selection and model fitting confined to the training set; model selection used TCGA-HNSC and the external model-selection cohort GSE42743 (*n* = 74 with overall survival), and the three independent test cohorts were scored without re-fitting (E-MTAB-8588, *n* = 83; GSE65858, *n* = 270; and GSE41613, *n* = 97). Key model genes were characterised by single-cell RNA sequencing (168,742 cells), spatial transcriptomics, and AI-guided screening of 249,455 compounds. **Results:** Thirty-one prognostic sialylation-associated DEGs defined an immunosuppressive subtype (C1) and an immune-active subtype (C2) with divergent survival. The SNI achieved a C-index of 0.88 in TCGA-HNSC, 0.68 in GSE42743, and 0.58–0.62 in the three independent test cohorts and remained independently prognostic after adjustment for age and other clinicopathological variables in multivariable analysis. SHAP analysis identified HSPH1 (risk-associated) and ST6GALNAC1 (protective) as principal contributors with opposing microenvironmental associations. **Conclusions:** Sialylation constitutes a distinct immunosuppressive axis in HNSCC, complementary to PD-1 blockade. The SNI provides a biologically anchored prognostic framework whose cross-platform transfer requires recalibration; putative candidate compounds require experimental validation.

## 1. Introduction

Head and neck squamous cell carcinoma (HNSCC) is the seventh most common cancer worldwide, with roughly 792,000 new cases and 424,000 deaths in 2020 alone [1]. It arises from the mucosal epithelium of the oral cavity, oropharynx, larynx, and hypopharynx, and its aetiology varies substantially by anatomical site. Tobacco and alcohol are the dominant risk factors for most subsites; high-risk human papillomavirus drives a biologically distinct oropharyngeal subtype with a generally more favourable prognosis [2]. For locally advanced disease, cisplatin-based chemoradiotherapy, combined with surgery where feasible, has been the standard of care for decades. Immune checkpoint blockade has changed this picture, at least in part. In the KEYNOTE-048 trial, pembrolizumab improved overall survival over cetuximab plus platinum-based chemotherapy in patients with PD-L1-positive recurrent or metastatic HNSCC [3], and perioperative pembrolizumab was subsequently shown to improve event-free survival in locally advanced resectable disease [4]. Yet the objective response rate to PD-1 monotherapy in unselected populations remains under 20% [5], and five-year overall survival has barely shifted over two decades. No validated biomarker reliably identifies patients most likely to benefit, and molecularly grounded therapeutic targets remain scarce. Better prognostic tools and a clearer understanding of the biology underlying immunotherapy failure are urgently needed.

Sialylation is a post-translational modification in which sialic acid residues are added to the terminal positions of glycoprotein and glycolipid chains by members of the sialyltransferase superfamily, including the ST6GAL, ST3GAL, and ST6GALNAC subfamilies [6,7]. In cancer, this modification is frequently and profoundly altered. Tumour cells accumulate a dense sialic acid-rich glycocalyx that shields them from immune surveillance and suppresses anticancer immunity by engaging inhibitory Siglec receptors on myeloid cells and natural killer cells [8]. This is not merely a passive barrier effect. Sialylated glycan ligands on tumour surfaces actively bind Siglec-7 and Siglec-9, triggering inhibitory signalling that reduces cytokine secretion and blocks immune-mediated killing—an effect demonstrated in prostate cancer and likely operative across tumour types [7]. Sialylated self-ligands on tumour cells can likewise engage Siglec receptors on tumour-infiltrating T cells, restraining effector function through a pathway parallel to PD-1 signalling [9]. Because this Siglec-mediated suppression is mechanistically orthogonal to the PD-L1/PD-1 axis, it can sustain immune suppression even while checkpoint blockade is applied—a plausible basis for the limited response rates to PD-1-directed therapy in HNSCC. Reciprocally, enzymatic removal of tumour-cell sialic acids enhances T-cell-mediated killing in preclinical tumour models [10,11,12], supporting sialylation blockade as a potential combination partner for immune checkpoint therapy. Sialylation also shapes tumour biology more broadly. In colorectal carcinoma, ST6GALNAC1 contributes to the synthesis of the sialyl-Tn (sTn) antigen, which is associated with tumour progression [13]. Comparable roles for sialylation in immune suppression and stromal remodelling have been reported in breast, prostate, and other solid tumours [6,7]. In HNSCC, a glycosylation-based prognostic model was recently described [14], but a systematic investigation of the sialylation-specific gene landscape—its prognostic relevance, molecular subtype associations, immune microenvironment correlates, cell-type-resolved expression patterns, and therapeutic potential—has not been carried out. This gap is notable, given that HNSCC tumours are often deeply immunosuppressive and respond poorly to PD-1 blockade.

Several technological advances now make a comprehensive investigation of this kind feasible. Single-cell RNA sequencing has substantially deepened our understanding of the HNSCC tumour microenvironment. It has identified cancer-associated fibroblast subsets that physically restrict CD8+ T-cell infiltration [15], mapped the stepwise transcriptional changes accompanying malignant progression [16], and revealed immune evasion strategies operative at the earliest stages of metastatic spread [17]. Spatial transcriptomics has added a tissue-level dimension. Studies have shown that tumour-core and tumour-edge transcriptional architectures are spatially conserved across patients and carry independent prognostic significance [18], and that the cellular trajectory landscape of the HNSCC ecosystem follows structured patterns across epithelial, stromal, and immune compartments [19]. When focused on a specific biological axis such as sialylation, these technologies can pinpoint which cell types drive pathway activity, resolve its spatial organisation within intact tissue, and link it to functional immune phenotypes. Machine learning has matured in parallel. Benchmarking large numbers of survival-modelling algorithm combinations can enable more systematic model selection, provided that performance is reported per cohort rather than as a single cross-cohort aggregate. At the therapeutic interface, AI-guided computational pipelines have redefined early drug discovery. Graph neural network-based drug–target interaction models [20], deep-learning pharmacokinetic and toxicological filters, AlphaFold2-guided structure-based docking [21], and molecular dynamics simulation can together screen hundreds of thousands of compounds and prioritise candidates with defined structural and pharmacological properties—a scale and speed inaccessible to conventional biochemical approaches.

In this study, we aimed to map the prognostic landscape of sialylation in HNSCC, to identify candidate genes through which sialylation may shape the tumour niche, and to translate these findings into candidate therapeutic interventions. To this end, we integrated transcriptomic data from seven GEO cohorts with TCGA-HNSC, intersecting differentially expressed genes with a curated 1204-gene sialylation compendium to define 156 sialylation-associated DEGs, of which 31 were prognostically significant. Consensus clustering of these 31 genes partitioned patients into a sialylation-enriched, immunosuppressive subtype (C1) and an immune-active subtype (C2) with divergent survival. Benchmarking 117 machine-learning algorithm combinations (nominally 101), we built the Sialylation Niche Index (SNI), a hybrid stepwise-Cox and random-survival-forest score that was an independent prognostic factor in multivariable analysis and was evaluated cohort by cohort across the training, model-selection, and three independent external test datasets. SHAP analysis identified ST6GALNAC1 and HSPH1 as the principal model contributors with opposing tumour microenvironment associations, and single-cell RNA sequencing of 168,742 cells and 10× Visium spatial transcriptomics resolved their cell-type distribution and spatial co-enrichment within the tumour niche. Finally, an AI-guided pipeline screened 249,455 compounds through dual-model drug–target interaction prediction, multi-criteria ADMET filtering, structure-based docking, and molecular dynamics simulation, nominating putative candidates against each target. This work thereby establishes a sialylation-centred prognostic framework for HNSCC, together with a putative mechanistic framework and a computationally grounded foundation for subsequent experimental and therapeutic development.

## 2. Methods

### 2.1. Data Sources

The following seven GEO transcriptomic datasets were used for differential expression analysis: GSE29330 [22] (*n* = 18), GSE30784 [23] (*n* = 212), GSE3292 [24] (*n* = 36), GSE42743 [25] (*n* = 103), GSE6791 [26] (*n* = 56), GSE7224 [27] (*n* = 13), and GSE9844 [28] (*n* = 38), totalling 476 samples with tumour and normal tissue (Table 1). The Cancer Genome Atlas HNSCC cohort (TCGA-HNSC; *n* = 501) served as the primary training cohort. GSE42743 (*n* = 74 with overall survival) was the external model-selection cohort. The following three independent external test cohorts with survival data were scored with the SNI model: E-MTAB-8588 [29] (Illumina HumanHT-12; *n* = 215, of which 83 with overall survival), GSE65858 [30] (*n* = 270), and GSE41613 [25] (*n* = 97; follow-up in months). A sialylation gene compendium of 1204 entries was downloaded from GeneCards (https://www.genecards.org/) using the keyword “Sialylation” on 24 April 2026, without additional filtering (Table S1). Single-cell RNA-seq data were obtained from GSE234933 [31] (168,742 cells, 52 samples after quality control). Spatial transcriptomics data were obtained from GSE181300 [32] (10× Visium, 8 HNSCC sections). All datasets are publicly available; no additional ethical approval was required.

**Figure 1:**
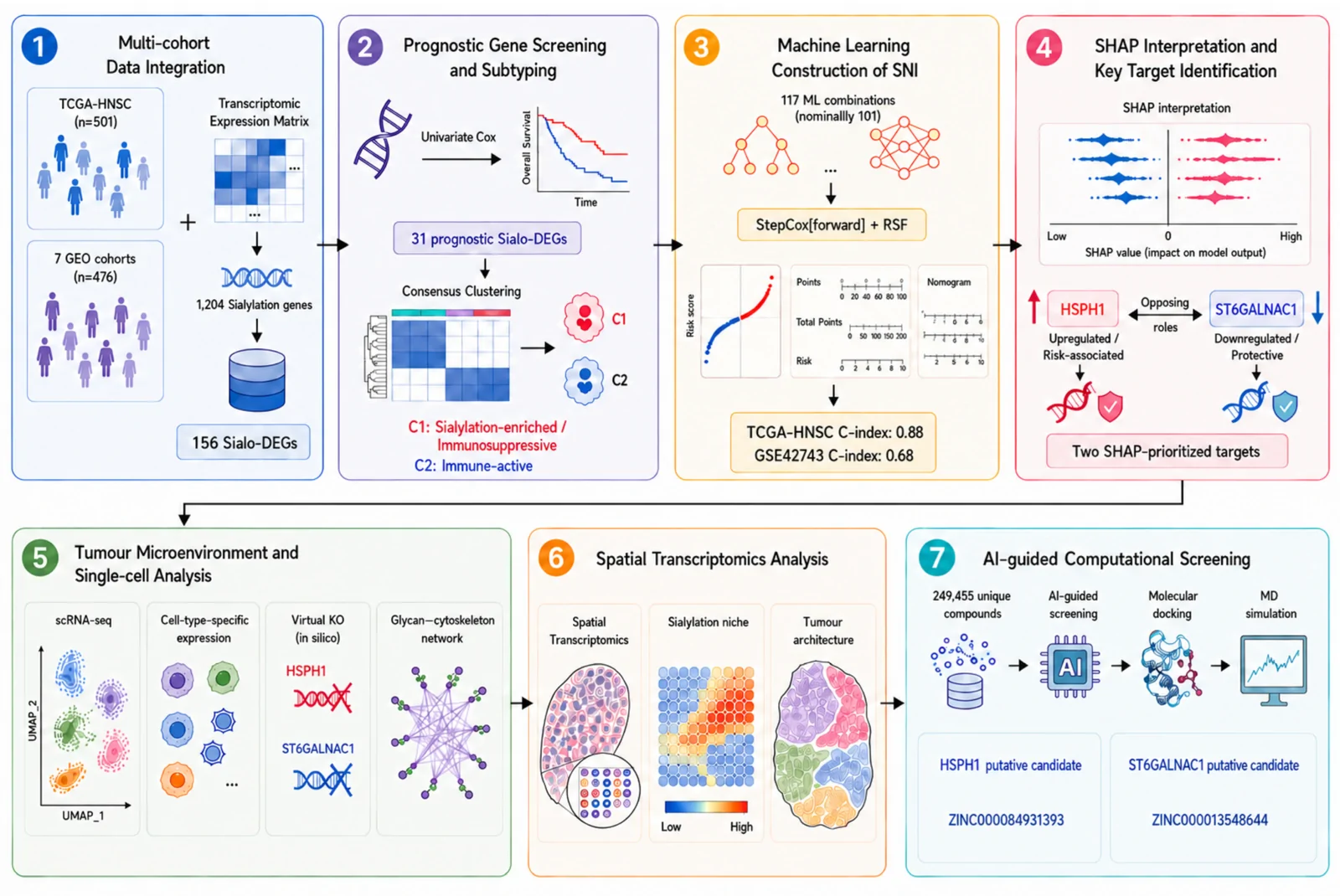
Workflow of this study.

Artificial intelligence tools were used in this study for the following purposes: GraphBAN for drug–target interaction prediction; ADMET-AI for pharmacokinetic and toxicological filtering; AlphaFold2 for protein structure prediction; and the fastshap package for Monte Carlo SHAP approximation. These tools were applied for the analyses listed above and are cited in the Methods and References sections accordingly. Large language model (LLM) assistance was used for drawing the overall workflow illustration (Figure 1).

### 2.2. Identification of Sialylation-Associated Prognostic Genes

Inter-dataset batch effects among the seven GEO microarray cohorts were removed using removeBatchEffect from the limma package; this correction was applied only for the differential-expression (DEG discovery) step. Model training used TCGA-HNSC alone, and the external model-selection cohort GSE42743 and the three independent external test cohorts were scored with the trained model, without cross-platform mixing. Differential expression between tumour and normal tissue was assessed with empirical Bayes moderation, retaining genes with adjusted *p* < 0.05 and |log_2_FC| > 1 (1293 DEGs). Intersection with the sialylation compendium yielded 156 Sialo-DEGs. Univariate Cox proportional hazards regression was applied to each Sialo-DEG in TCGA-HNSC, excluding genes missing in more than 25% of samples; genes with *p* < 0.05 were retained, yielding 31 prognostic Sialo-DEGs. Survival analyses used complete-case samples with available overall-survival information; no global outlier removal was applied. Bootstrap stability of Cox coefficients was assessed with 1000 resamples.

GO (biological process and cellular component ontologies) and KEGG pathway enrichment of the 31 prognostic genes were performed with clusterProfiler and org.Hs.eg.db, with Benjamini–Hochberg FDR correction; up to ten terms per regulation direction were displayed for each ontology/database, where available. Somatic mutation data were visualised as an oncoplot. Chromosomal coordinates (GRCh38) were retrieved from Ensembl via biomaRt and displayed as a circos plot.

### 2.3. Molecular Subtyping and Immune Landscape

Unsupervised consensus clustering (ConsensusClusterPlus) was applied to the 31-gene expression profiles using hierarchical clustering with Pearson distance, 1000 bootstrap iterations, and 80% item resampling. Optimal k = 2 was selected by cophenetic correlation and proportion of ambiguously clustered pairs (PAC); survival differences were assessed by Kaplan–Meier analysis with log-rank test. GSEA of C1-versus-C2 differential expression (genes ranked by limma t-statistic) was performed against KEGG and GO gene sets using clusterProfiler, with nominal *p* < 0.05 as the significance threshold.

Tumour purity, immune score, and stromal score were estimated with the ESTIMATE algorithm. Expression of ten immune checkpoint genes (CD274, CTLA4, HAVCR2, IGSF8, CD1B, LAG3, PDCD1, PDCD1LG2, SIGLEC15, and TIGIT) was compared between clusters by Wilcoxon rank-sum test. TIDE analysis was performed using ssGSEA with robust scaling (median ± MAD) to score cancer-immunity cycle steps.

### 2.4. Machine-Learning Model and SNI Construction

One hundred and seventeen algorithm combinations (nominally 101) implemented in the Mime1 framework [33] were benchmarked for survival prediction, with TCGA-HNSC as the training cohort and GSE42743 (n = 74 with overall survival) as the external model-selection cohort (Table S2). The model-selection criterion was the highest mean C-index across these two cohorts. StepCox[forward] + RSF achieved this maximum tied with the RSF model alone, and the hybrid was retained. Candidate features were the 31 prognostic Sialo-DEGs; within the framework these were further filtered by univariate Cox regression in TCGA-HNSC at unicox_p_cutoff = 0.01, yielding the 11-gene signature. The combination of StepCox[forward] (forward stepwise selection by AIC) and random survival forest (RSF; nodesize = 10, seed = 2026) achieved the highest mean C-index, tied with the RSF model alone, and was retained as the final model. The reported C-indices are apparent in-sample concordance statistics, i.e., the concordance of a univariate Cox regression of each cohort’s risk score, with larger risk scores indicating worse expected survival. The SNI is the random-survival-forest risk score; for the multivariable Cox and nomogram analyses the score was z-standardised within TCGA-HNSC (per-SD hazard ratios), whereas the external-cohort Cox models used the raw score (per-unit hazard ratios), and the per-patient ranking displays use z-standardised values; 10-fold cross-validation was used only within the hyperparameter tuning of individual learners, and the final pipeline itself was not cross-validated. As an uncertainty measure, a 1000-resample bootstrap of the training C-index yielded a 95% confidence interval of 0.860–0.894. The resulting continuous score was designated the Sialylation Niche Index (SNI); patients were stratified into high- and low-risk groups using the median risk score within each cohort as the cut-off (a cohort-specific threshold used for within-cohort risk-stratification display rather than fixed-threshold external validation). Model performance was assessed by C-index and time-dependent AUC at 1, 2, and 3 years. Array-based expression data (GSE42743) were annotated using AnnotationDbi, with multiple probes collapsed by arithmetic mean. For multi-cohort scoring, candidate genes were intersected to those present in every scored cohort (TGFB1 and CXCL8 are absent from some external matrices, shrinking the candidate set from 31 to 29); both genes have TCGA-HNSC univariate Cox *p* > 0.01 and are removed by the internal filter, so the 11-gene signature and all reported model values are unaffected.

### 2.5. Nomogram, SHAP Interpretation, and Target Selection

Univariate and multivariable Cox regression incorporating age, AJCC stage, T category, N category, and tumour grade, together with SNI, was performed to assess the prognostic independence of SNI. A clinical nomogram integrating age and SNI was constructed with the rms package and calibrated at 1, 2, and 3 years using 500 bootstrap resamples. Decision curve analysis was performed using rmda (threshold range 0–1).

SHAP (SHapley Additive exPlanations) was applied to both model components. For the StepCox component, SHAP values were derived from the linear predictor (type = “lp”). For the RSF component, SHAP values were estimated via Monte Carlo approximation. Final gene selection required genes to satisfy the following two criteria simultaneously: ranking in the top 5 by mean absolute SHAP value AND exceeding the median interquartile range (IQR = Q_75_ − Q_25_) of SHAP values across all genes. This dual criterion, applied to the RSF SHAP matrix, identified ST6GALNAC1 and HSPH1 as the two priority targets. To assess the robustness of this selection, the thresholds were varied over top-K ∈ {3, 5, and 10} and IQR cut-offs ∈ {none, median, and 75th percentile} (Table S3); ST6GALNAC1 and HSPH1 were retained under all nine settings.

### 2.6. Target Gene Characterisation and Immune Microenvironment

Differential expression of HSPH1 and ST6GALNAC1 was validated by Wilcoxon rank-sum test across TCGA-HNSC, E-MTAB-8588, and GSE42743. Partial correlations between the two genes were computed after adjusting for age and tumour purity. Immune cell composition was estimated from TCGA-HNSC bulk RNA-seq using the following six algorithms in the immunedeconv package: TIMER, CIBERSORT, xCell, MCP-counter, EPIC, and QUANTISEQ. Associations with immune cell fractions, ESTIMATE scores, immune checkpoint expression (CD274), and microsatellite instability score were quantified by Spearman rank correlation with Benjamini–Hochberg FDR correction. Pan-cancer expression differences were evaluated across available TCGA cohorts by Wilcoxon rank-sum test.

### 2.7. Single-Cell RNA-Seq and Virtual Gene Knockout

scRNA-seq data from GSE234933 (168,742 cells, 52 samples) were processed and cell types annotated using canonical marker genes in Seurat. Cluster marker genes were identified with FindAllMarkers (min.pct = 0.25, logFC. Threshold = 0.25, Wilcoxon test); GO biological process enrichment was performed with clusterProfiler (pvalueCutoff = 0.5, top 5 terms per cell type). Expression patterns were visualised using ClusterGVis.

Virtual gene knockout of HSPH1 and ST6GALNAC1 was performed in isolated tumour epithelial cells using scTenifoldKnk. The 1000 tumour cells with the highest mean expression of each target gene were selected. Gene regulatory networks were constructed from nNet = 10 sub-networks, each built from nCells = 250 randomly sampled cells. Downstream perturbation was quantified as Euclidean distance between the original and knockout networks, converted to Z-scores and log_2_ fold changes.

### 2.8. Spatial Transcriptomics

Spatial transcriptomics data from GSE181300 (8 HNSCC Visium sections) were processed in Seurat v4. Expression matrices were normalised using SCTransform (2000 variable features); dimensionality reduction used PCA (top 30 components) followed by UMAP. Spots were clustered at resolution = 1.0 (Louvain algorithm) and annotated by canonical marker expression as follows: malignant epithelial cells (KRT5, KRT6A, KRT8, and EPCAM), T cells (CD2, CD3D, CD3E, CD3G, CD7, and CD8A), B cells (CD79A, CD79B, and CD19), fibroblasts (FAP, COL1A2), endothelial cells (PECAM1, VWF), and myocytes (ACTA1, ACTA2, ACTN2, MYL2, and MYH2). Spatial co-enrichment of HSPH1 and ST6GALNAC1 with annotated cell-type zones was assessed by the following two complementary methods: Fisher’s exact test (spot-level co-occurrence) and a nearest-neighbour (NN) distance permutation test using k = 6 spatial neighbours (FNN package) with 500 permutations to derive empirical *p*-values and enrichment odds ratios.

### 2.9. Computational Drug Screening

A ZINC compound library containing 249,455 unique molecules (after SMILES deduplication) was pre-filtered by Lipinski’s Rule of Five and PAINS criteria, retaining 237,296 compounds. Protein sequences for HSPH1 (UniProt Q92598) and ST6GALNAC1 (UniProt Q9NSC7) were retrieved from UniProt. AlphaFold2 structural models (AF-Q92598-F1; AF-Q9NSC7-F1) from the AlphaFold Protein Structure Database were used as docking receptors; binding pockets were defined by coordinate transfer from homologous crystal structures in the Protein Data Bank: HSPH1 from PDB 3FE1 (ADP-bound; local pocket RMSD 1.060 Å) and ST6GALNAC1 from PDB 6APL (ligand C5P; local pocket RMSD 0.297 Å).

Drug–target interaction prediction was performed with GraphBAN, which encodes molecular graphs (ChemBERTa) and protein sequences (ESM-1b) via a graph-based bilinear attention network. Two independently trained GraphBAN models (BioSNAP and KIBA datasets) were applied to each target, and per-compound mean scores were used for ranking. Pharmacokinetic and toxicological filtering used ADMET-AI, retaining compounds with AMES < 0.25, Carcinogens_Lagunin < 0.25, ClinTox < 0.25, DILI < 0.25, hERG < 0.25, Bioavailability_Ma > 0.5, Half_Life_Obach > 0, PPBR_AZ > 50%, and logP 1–3, yielding 164 candidates per target.

Structure-based docking of the top 20 ADMET-ranked candidates was performed with AutoDock Vina v1.2.5 (exhaustiveness = 32, num_modes = 9, and energy_range = 4 kcal/mol; docking box 20 × 20 × 20 Å^3^). Ligands were prepared with Meeko v0.7.1, RDKit v2026.03.1, and OpenBabel v3.1.1. Molecular dynamics simulations of the two docked complexes were conducted with GROMACS v2025.4 using the amber99sb-ildn force field (AmberTools v24.8) and GAFF2 ligand parameters (ACPYPE v2023.10.27), in explicit TIP3P water with periodic boundary conditions. The protocol comprised steepest descent energy minimization (50,000 steps; tolerance 1000 kJ mol^−1^ nm^−1^), NVT equilibration (100 ps, 298.15 K, V-rescale thermostat, and position restraints), NPT equilibration (100 ps, 1.0 bar, and C-rescale barostat), and 100 ns production MD (dt = 0.002 ps). Trajectories were analysed for backbone RMSD, RMSF, radius of gyration, SASA, protein–ligand contact counts (cutoff 0.45 nm), minimum protein–ligand distance, and hydrogen bond statistics; hydrogen bonds were computed with MDAnalysis 2.10.0 using default geometric criteria (donor–acceptor distance ≤ 0.30 nm and donor–hydrogen–acceptor angle ≥ 150°), because gmx hbond is inapplicable to the ligand topologies used in these simulations.

## 3. Results

### 3.1. Identification of Sialylation-Associated Prognostic Genes in Head and Neck Squamous Cell Carcinoma

**Figure 2:**
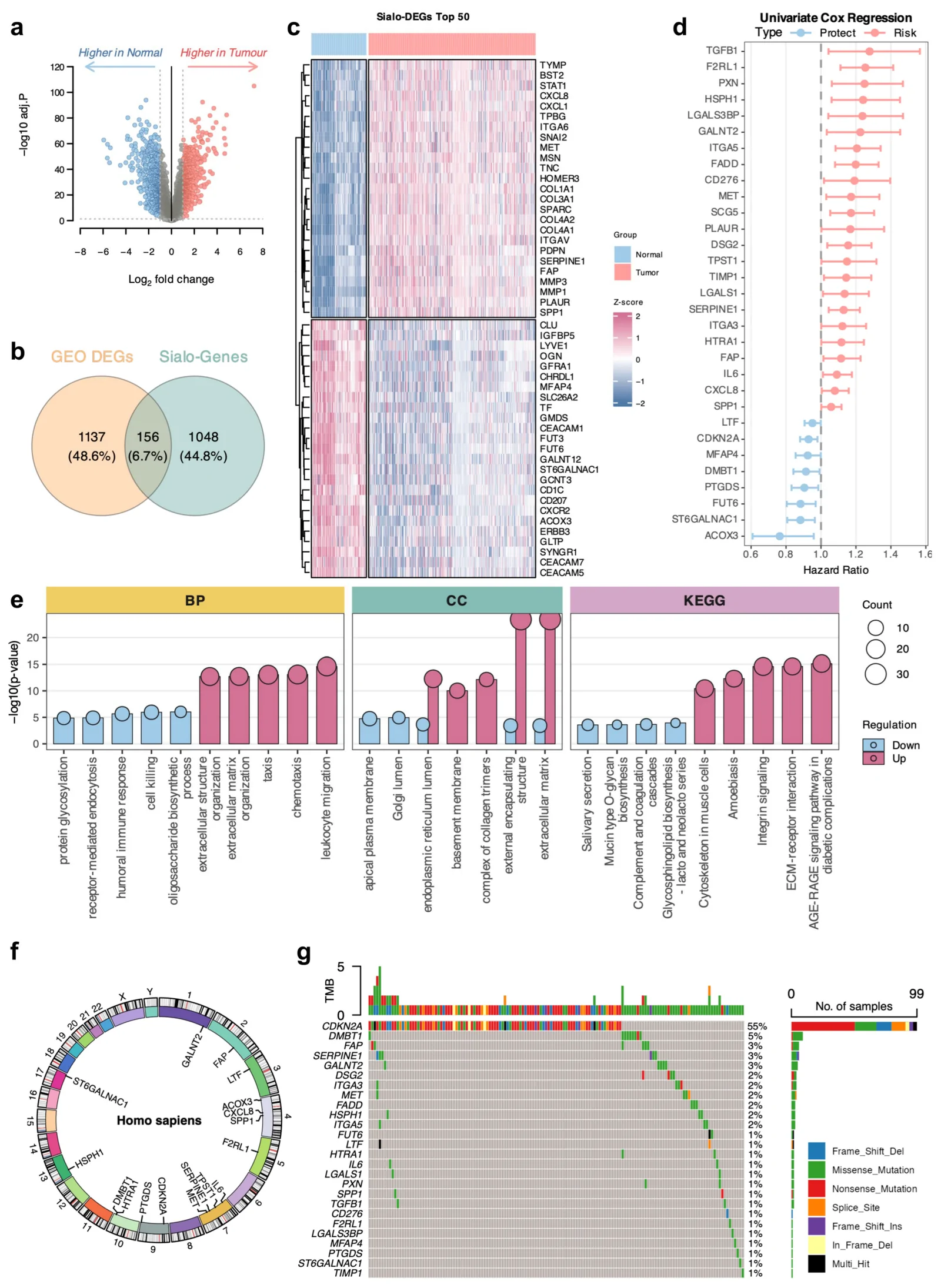
Identification of sialylation-associated differentially expressed genes with prognostic significance in HNSCC. (**a**) Volcano plot of differentially expressed genes (DEGs) between tumour and normal tissue across the seven GEO cohorts (1293 DEGs at adjusted *p* < 0.05 and |log2FC| > 1; red, higher in tumour; blue, higher in normal). (**b**) Venn diagram of the intersection between GEO DEGs and the 1204-gene sialylation compendium, yielding 156 sialylation-associated DEGs (Sialo-DEGs). (**c**) Unsupervised hierarchical clustering heatmap of the top 50 Sialo-DEGs across samples of the seven cohorts, separating tumour from normal tissue (Z-score). (**d**) Univariate Cox regression forest plot of the 31 prognostic Sialo-DEGs in TCGA-HNSC, with hazard ratios (HRs) and 95% confidence intervals (CIs); red, risk-associated genes; blue, protective genes. (**e**) Gene Ontology (biological process and cellular component) and KEGG pathway enrichment of the 31 prognostic genes (up to ten terms per regulation direction for each ontology/database, where available). (**f**) Circos plot showing the chromosomal distribution of the 31 genes (GRCh38). (**g**) Oncoplot of somatic mutation frequencies of the 31 genes in TCGA-HNSC, with tumour mutation burden per sample.

The overall workflow of this study is illustrated in Figure 1. To systematically identify sialylation-related genes with prognostic relevance in HNSCC, we integrated transcriptomic data from the following seven publicly available Gene Expression Omnibus (GEO) datasets comprising 476 samples in total: GSE29330 (*n* = 18), GSE30784 (*n* = 212), GSE3292 (*n* = 36), GSE42743 (*n* = 103), GSE6791 (*n* = 56), GSE7224 (*n* = 13), and GSE9844 (*n* = 38), encompassing tumour and normal tissue samples. After inter-dataset batch correction, differential expression analysis within the same framework identified 1293 differentially expressed genes (DEGs) significantly dysregulated between tumour and normal tissues (Figure 2a). To restrict the analysis to sialylation biology, these candidates were intersected with a curated sialylation gene compendium comprising 1204 entries compiled from GeneCards, yielding 156 sialylation-associated DEGs (Sialo-DEGs; Figure 2b). Unsupervised hierarchical clustering confirmed that the expression profiles of these 156 Sialo-DEGs clearly distinguished tumour from normal tissue across all seven cohorts (Figure 2c; top 50 shown).

To assess prognostic significance, univariate Cox proportional hazards regression was performed using overall survival data from The Cancer Genome Atlas HNSCC cohort (TCGA-HNSC; *n* = 501). Thirty-one genes reached statistical significance (Figure 2d). Risk-associated genes included HSPH1 (hazard ratio [HR] 1.24, 95% confidence interval [CI] 1.06–1.45, and *p* = 0.007), SERPINE1 (HR 1.13, *p* = 0.002), ITGA5 (HR 1.21, *p* < 0.001), FADD (HR 1.20, *p* < 0.001), and F2RL1 (HR 1.25, *p* < 0.001). Genes associated with favourable prognosis included ST6GALNAC1 (HR 0.88, 95% CI 0.81–0.96, and *p* = 0.006), CDKN2A (HR 0.93, *p* = 0.007), LTF (HR 0.95, *p* = 0.044), and PTGDS (HR 0.90, *p* = 0.020). Gene Ontology (GO) and Kyoto Encyclopaedia of Genes and Genomes (KEGG) pathway enrichment analysis of the 31 prognostic genes highlighted protein glycosylation, extracellular biosynthetic processes, and extracellular matrix (ECM)–receptor interaction as the most significantly enriched functional categories (Figure 2e), consistent with established roles of sialylation in shaping the tumour glycocalyx and modulating immune cell recognition. Chromosomal mapping and somatic mutation frequency analysis further demonstrated that these 31 genes were distributed across multiple chromosomes with heterogeneous mutation burden in TCGA-HNSC (Figure 2f–g).

### 3.2. Sialylation Gene Signatures Define Immunologically Distinct Molecular Subtypes with Divergent Clinical Outcomes

**Figure 3:**
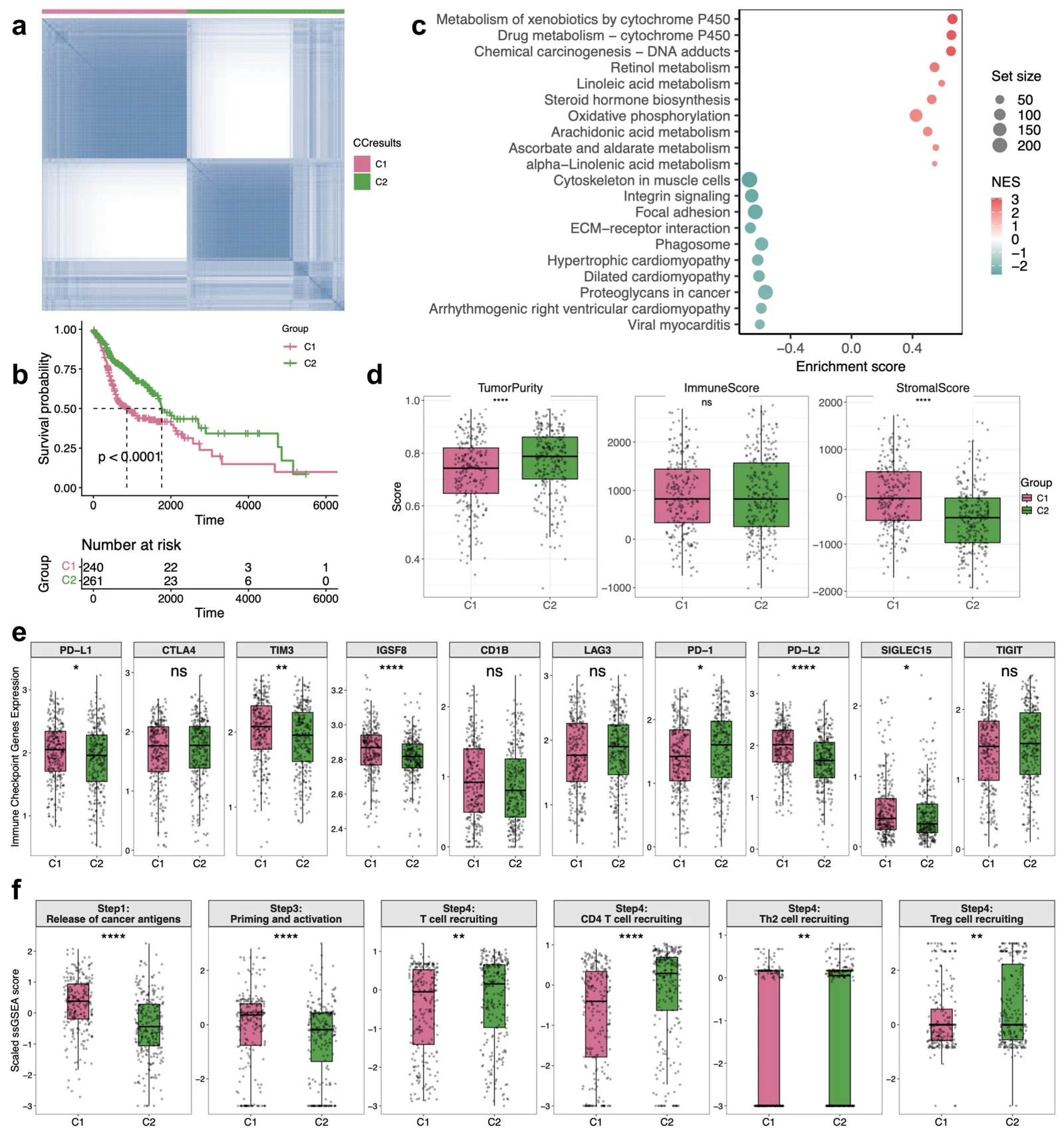
Sialylation gene expression defines immunologically distinct molecular subtypes with divergent survival outcomes. (**a**) Consensus clustering matrix of the 31 prognostic Sialo-DEGs in TCGA-HNSC, partitioning patients into two stable clusters (C1 and C2; k = 2). (**b**) Kaplan–Meier curves of overall survival for C1 (*n* = 240) and C2 (*n* = 261) (log-rank *p* < 0.0001). (**c**) GSEA dot plot of the top enriched KEGG terms between C1 and C2: focal adhesion, ECM–receptor interaction and integrin signalling enriched in C1, and xenobiotic metabolism by cytochrome P450 and drug metabolism via cytochrome P450 enriched in C2 (enrichment score, NES and set size shown). (**d**) ESTIMATE-based scores in C1 vs. C2, showing lower tumour purity (Wilcoxon *p* = 8.6 × 10^−5^) and higher stromal scores (*p* = 1.9 × 10^−13^) in C1, with no significant difference in immune scores (*p* = 0.99). (**e**) Expression of ten immune checkpoint genes in C1 vs. C2 (PD-L1, CTLA4, TIM3, IGSF8, CD1B, LAG3, PD-1, PD-L2, SIGLEC15, TIGIT; Wilcoxon rank-sum test), showing elevated PD-L1, TIM3, IGSF8, PD-L2 and SIGLEC15 in C1 and higher PD-1 in C2. (**f**) TIDE-based cancer-immunity cycle step scores (scaled ssGSEA score; Wilcoxon test): C1 scored higher at cancer antigen release and T-cell priming/activation steps, whereas C2 showed higher scores in T-cell recruiting steps including CD4, Th2 and Treg functions. Asterisks indicate statistical significance: * *p* < 0.05; ** *p* < 0.01; **** *p* < 0.0001.

Having identified 31 prognostic Sialo-DEGs, we investigated whether their combined expression could stratify TCGA-HNSC patients into biologically and clinically distinct subgroups. Unsupervised consensus clustering was applied to the expression profiles of these 31 genes across TCGA-HNSC, using hierarchical clustering with Pearson distance, 1000 bootstrap iterations, and 80% item resampling. Cluster stability was optimal at k = 2, partitioning patients into groups designated C1 and C2 (Figure 3a). Kaplan–Meier analysis revealed significantly inferior overall survival in C1 relative to C2 (Figure 3b), indicating that differential sialylation-related gene activity corresponds to distinct survival outcomes.

Gene set enrichment analysis (GSEA) of differentially expressed genes between C1 and C2 identified enrichment of focal adhesion, ECM–receptor interaction, and integrin signalling in C1 (Figure 3c), suggesting an invasive, stromal-remodelling phenotype, whereas xenobiotic metabolism by cytochrome P450 and drug metabolism via cytochrome P450 were enriched in C2. ESTIMATE-based deconvolution confirmed lower tumour purity and higher stromal scores in C1, with no significant difference in immune scores between the two groups (Figure 3d). Analysis of immune checkpoint gene expression showed that C1 was characterised by markedly elevated programmed death-ligand 1 (PD-L1; CD274), TIM3, IGSF8, PD-L2, and SIGLEC15, whereas programmed cell death protein 1 (PD-1; PDCD1) was more highly expressed in C2 (Figure 3e). SIGLEC15 has been identified as an immune checkpoint that suppresses T-cell proliferation independently of PD-L1 [34], and its co-upregulation with PD-L1 and PD-L2 in C1 indicates a multi-layered immunosuppressive programme.

We further applied Tumour Immune Dysfunction and Exclusion (TIDE), a computational framework that models immune evasion mechanisms by integrating signals across the cancer antigen release, T-cell priming, and effector recruitment steps of the cancer-immunity cycle [35]. C1 patients scored higher at the stages of cancer antigen release and priming/activation, whereas C2 patients showed higher scores in T-cell recruiting steps including CD4, Th2, and Treg functions (Figure 3f). Collectively, these findings characterise C1 as a sialylation-enriched, stromal-dense subtype with concurrent upregulation of multiple immune checkpoints and reduced T-cell effector recruitment and motivate the construction of a continuous, patient-level prognostic index to capture this immunological heterogeneity.

### 3.3. Construction and External Evaluation of the Sialylation Niche Index Using an Ensemble Machine-Learning Framework

**Figure 4:**
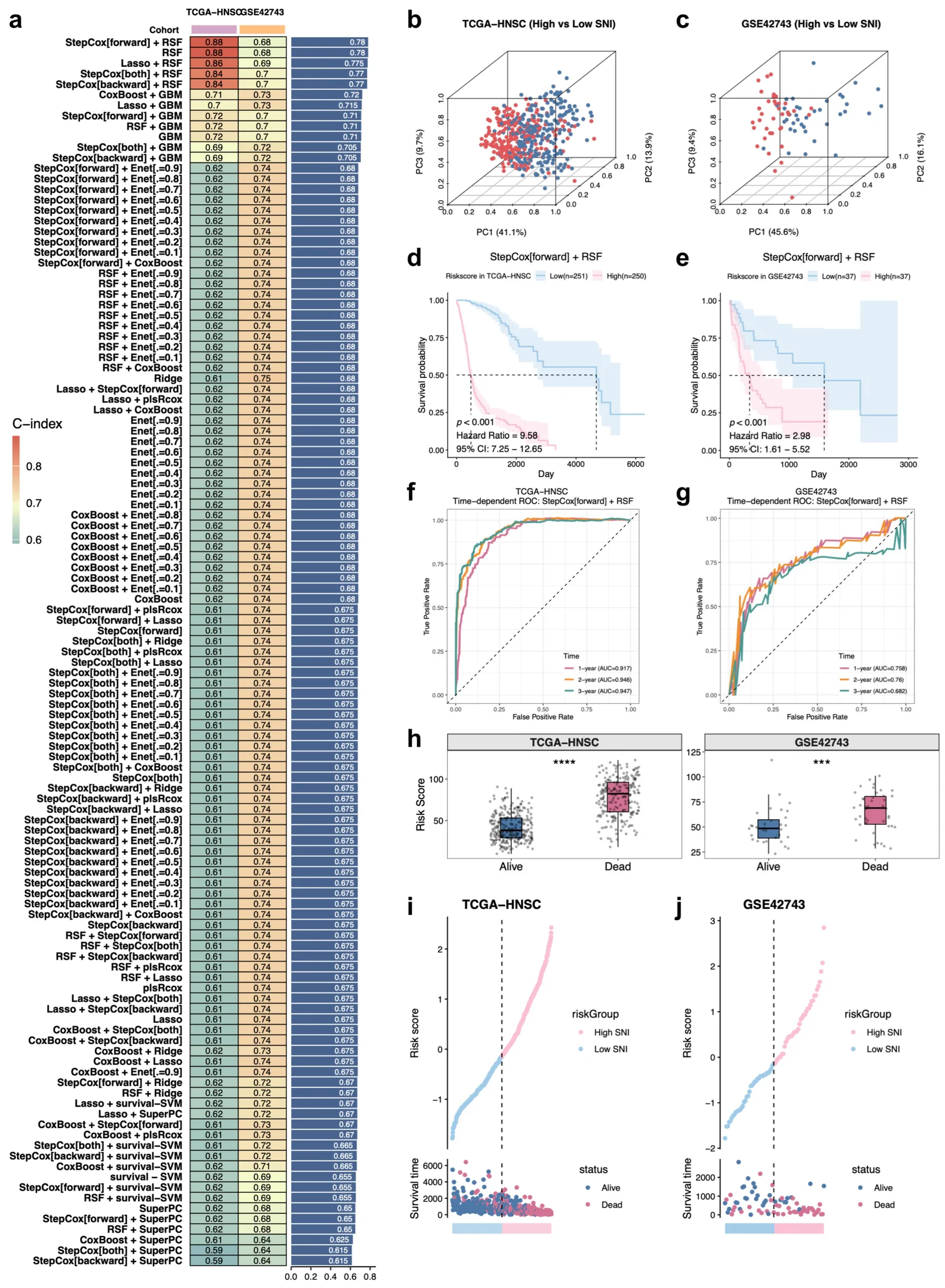
Construction and external evaluation of the Sialylation Niche Index in the TCGA-HNSC training cohort and the GSE42743 external model-selection cohort. (**a**) Benchmarking of the 117 machine-learning algorithm combinations (nominally 101) by C-index in TCGA-HNSC (training) and GSE42743 (external model selection); StepCox[forward] + RSF achieved the highest mean C-index (0.88 and 0.68 per cohort; mean 0.78), tied with the RSF model alone, and was selected as the final model. (**b**) Three-dimensional principal component analysis of SNI signature-gene expression separating high- and low-risk patients in TCGA-HNSC (PC2 13.9%, PC3 9.7%). (**c**) The same analysis in GSE42743 (PC2 16.1%, PC3 9.4%). (**d**) Kaplan–Meier curves of overall survival for high- vs. low-SNI patients in TCGA-HNSC (HR 9.58, 95% CI 7.25–12.65, *p* < 0.001). (**e**) Kaplan–Meier curves in GSE42743 (HR 2.98, 95% CI 1.61–5.52, *p* < 0.001). (**f**) Time-dependent ROC curves of the SNI at 1, 2 and 3 years in TCGA-HNSC (AUC 0.917, 0.946 and 0.947). (**g**) Time-dependent ROC curves in GSE42743 (AUC 0.758, 0.760 and 0.682). (**h**) SNI risk scores in patients alive vs. deceased during follow-up in TCGA-HNSC and GSE42743 (Wilcoxon rank-sum test; significance indicated by stars). (**i**) Per-patient z-standardised SNI risk scores, survival time and risk-group assignment in TCGA-HNSC, ranked by SNI (high SNI, red; low SNI, blue). (**j**) The same z-standardised ranking display in GSE42743. Per-cohort survival and discrimination results for the external model-selection cohort GSE42743 and the three independent external test cohorts are provided in Figures S1–S3 and Table 2. Asterisks indicate statistical significance: *** *p* < 0.001; **** *p* < 0.0001.

Building on the two-cluster stratification, we sought to develop a continuous, patient-level prognostic score that could quantify the sialylation niche and be evaluated cohort by cohort in independent data. Using the 31 prognostic Sialo-DEGs as candidate features, we systematically evaluated 117 machine-learning algorithm combinations (nominally 101) implemented in the Mime1 framework, with TCGA-HNSC as the training cohort and GSE42743 as the external model-selection cohort; the model-selection criterion was the highest mean C-index across these two cohorts. The combination of stepwise Cox regression with forward selection (StepCox[forward]) and random survival forest (RSF) achieved the highest mean C-index, tied with the RSF model alone, and was retained as the final model (Figure 4a). In this hybrid framework, StepCox[forward] performs initial variable selection based on linear hazard associations, and the resulting gene subset is then used to train an RSF model, which captures non-linear survival dependencies. The resulting score was designated the Sialylation Niche Index (SNI). Its performance is reported per cohort (Table 2): the C-index was 0.88 in the training cohort TCGA-HNSC (n = 501; 1000-resample bootstrap 95% CI 0.860–0.894) and 0.68 in the external model-selection cohort GSE42743 (n = 74), and 0.62 in E-MTAB-8588 (n = 83), 0.58 in GSE65858 (n = 270), and 0.62 in GSE41613 (n = 97), the three independent external test cohorts. Time-dependent AUCs at 1, 2, and 3 years were 0.917, 0.946, and 0.947 in TCGA-HNSC and 0.758, 0.760, and 0.682 in GSE42743 (Figure 4f,g), and 0.568, 0.635, and 0.662 in E-MTAB-8588, 0.634, 0.608, and 0.587 in GSE65858, and 0.764, 0.711, and 0.680 in GSE41613 (computed at 12, 24, and 36 months; Figure S1). Performance was therefore strongest in the training cohort and the external model-selection cohort and moderate in the three independent external test cohorts, where cross-platform transfer attenuated but did not abolish the prognostic signal.

Three-dimensional principal component analysis (PCA) of SNI signature-gene expression demonstrated clear separation of high- and low-SNI patients in both cohorts (Figure 4b,c). Kaplan–Meier analysis showed that high SNI was strongly associated with worse overall survival in TCGA-HNSC (HR = 9.58, 95% CI 7.25–12.65, and *p* < 0.001; Figure 4d) and in GSE42743 (HR = 2.98, 95% CI 1.61–5.52, and *p* < 0.001; Figure 4e). SNI scores were significantly higher in patients who died during follow-up than in survivors in both cohorts (Figure 4h), and individual patient survival time and outcome tracked consistently with SNI values across both datasets (Figure 4i,j), supporting the prognostic signal of the index in these cohorts.

As a benchmark against established clinical variables, the SNI was compared with a model built from conventional clinical features (age, AJCC stage, T category, N category, tumour grade, and sex): in the complete-case subset of TCGA-HNSC (n = 457), the clinical-only model reached a C-index of 0.604, the SNI alone 0.876, and the combined clinical-plus-SNI model 0.879, indicating that the SNI captures prognostic information beyond the conventional variables, whereas adding clinical variables to the SNI does not materially improve discrimination. To aggregate the external evidence, the risk scores of the four external cohorts were stacked (n = 524) and analysed with a cohort-stratified Cox model: higher SNI was associated with worse overall survival in every external cohort, with per-cohort hazard ratios per unit risk score of 1.023 (GSE42743; 95% CI 1.010–1.037), 1.016 (E-MTAB-8588; 1.005–1.027), 1.011 (GSE65858; 1.001–1.020), and 1.018 (GSE41613; 1.002–1.033), and the stratified pooled estimate was HR = 1.016 per unit risk score (95% CI 1.010–1.021, *p* = 1.95 × 10^−7^), with a pooled C-index of 0.60. The dichotomised Kaplan–Meier hazard ratios differed in magnitude between cohorts (HR = 9.58 in TCGA-HNSC vs. HR = 2.98 in GSE42743) but were directionally consistent: in every cohort the high-SNI group had the worse survival (Figure S2). The difference in magnitude reflects the cohort-specific median cut-off, cohort composition, and platform differences rather than a qualitative inconsistency; for cross-cohort comparison of the continuous score we therefore rely on the per-cohort C-index, which is rank-based and does not depend on the dichotomisation cut-off. Because the pooled statistics mix platforms and follow-up conventions, they should be read as aggregate directional evidence rather than a recalibrated clinical score.

### 3.4. SNI as an Independent Prognostic Factor, Clinical Nomogram Development, and SHAP-Based Feature Interpretation

**Figure 5:**
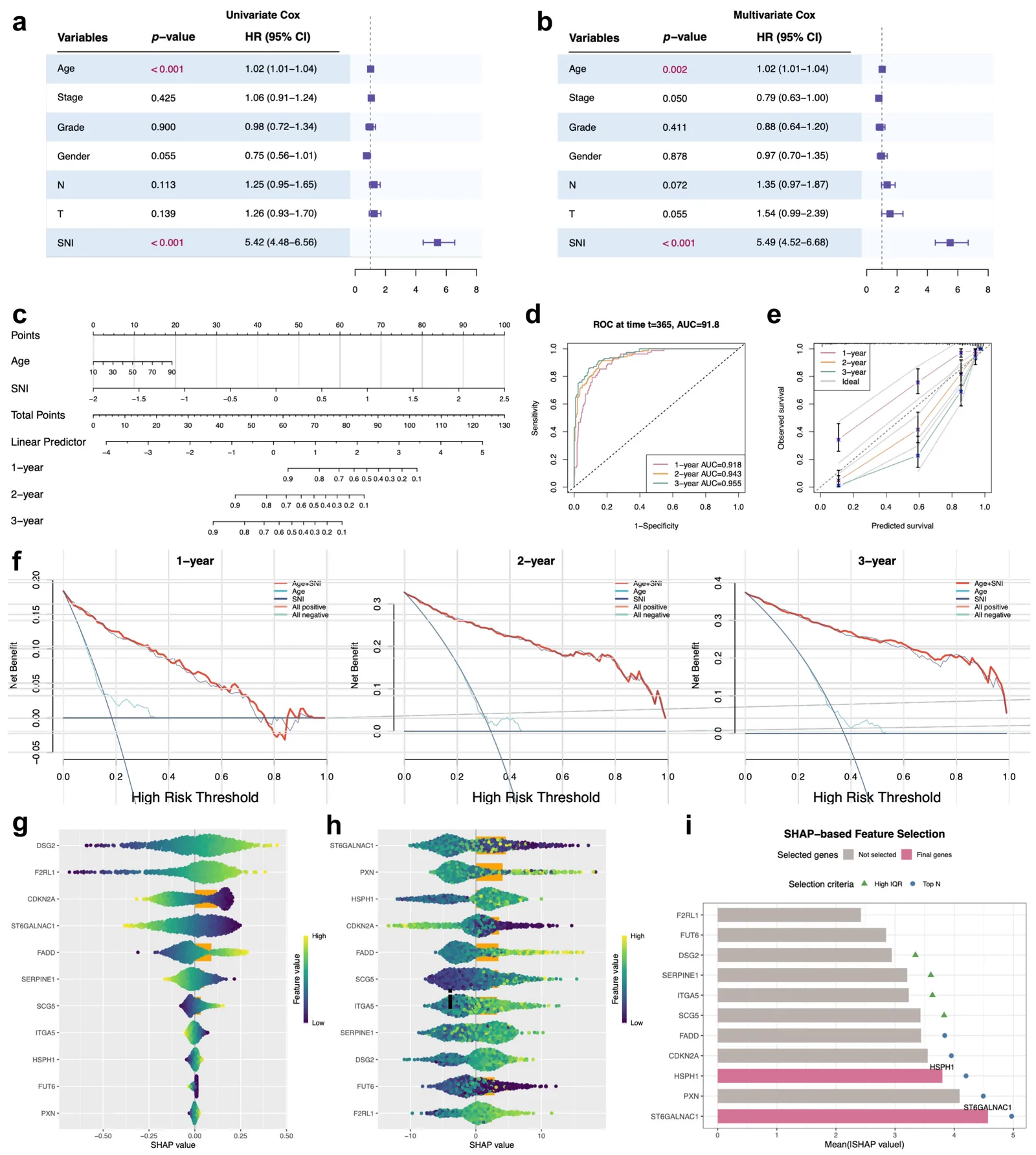
SNI as an independent prognostic factor: nomogram, calibration, and SHAP-based feature interpretation. (**a**) Univariate Cox regression of age, stage, grade, gender, N category, T category and SNI in TCGA-HNSC; only age (HR 1.02, 95% CI 1.01–1.04, *p* < 0.001) and SNI (HR 5.42, 95% CI 4.48–6.56, *p* < 0.001) were significant. (**b**) Multivariable Cox regression confirming age (HR 1.02, 95% CI 1.01–1.04, *p* = 0.002) and SNI (HR 5.49, 95% CI 4.52–6.68, *p* < 0.001) as independent prognostic factors. (**c**) Nomogram integrating age and z-standardised SNI for predicting 1-, 2- and 3-year overall survival. (**d**) Time-dependent ROC curves of the nomogram at 1, 2 and 3 years (AUC 0.918, 0.943 and 0.955). (**e**) Calibration curves of predicted vs. observed survival at 1, 2 and 3 years, with the ideal reference line. (**f**) Decision curve analysis at 1, 2 and 3 years showing the net benefit of the age-plus-SNI model compared with age alone, SNI alone, treat-all and treat-none strategies. (**g**) SHAP beeswarm plot of per-sample feature contributions from the StepCox component of the SNI model. (**h**) SHAP beeswarm plot from the RSF component (SHAP values estimated by Monte Carlo approximation with 200 iterations). (**i**) SHAP-based feature selection: genes ranked by mean |SHAP| value, with the top-5 (Top N) and high-IQR criteria indicated; genes satisfying both criteria (ST6GALNAC1 and HSPH1) were designated final priority targets.

To assess whether SNI provides prognostic information independent of clinicopathological variables, we incorporated age, AJCC stage, T category, N category, and tumour grade into univariate Cox regression. Only age (HR = 1.02, 95% CI 1.01–1.04, and *p* < 0.001) and SNI (HR = 5.42, 95% CI 4.48–6.56, and *p* < 0.001) reached significance (Figure 5a). Multivariable Cox regression confirmed both as independent predictors, with age retaining HR = 1.02 (95% CI 1.01–1.04) and SNI retaining HR = 5.49 (95% CI 4.52–6.68; Figure 5b). A nomogram integrating age and SNI was constructed (Figure 5c) and demonstrated 1-, 2-, and 3-year AUC values of 0.918, 0.943, and 0.955, respectively (Figure 5d). Calibration curves showed good agreement between predicted and observed survival at the three time points, indicating apparent calibration within the TCGA-HNSC development cohort (Figure 5e), and decision curve analysis indicated model-estimated net benefit of the age-plus-SNI model over the alternative strategies across a range of threshold probabilities within the development cohort (Figure 5f).

To identify the specific genes within the SNI model responsible for its predictive performance, we applied SHapley Additive exPlanations (SHAP) analysis [36], a game-theory-based framework that decomposes model predictions into additive per-feature contributions, enabling global feature ranking and sample-level interpretability. SHAP was applied separately to both model components: the StepCox component yielded SHAP values representing log-risk contributions under the Cox proportional hazards framework (Figure 5g), while for the RSF component, SHAP values were estimated via Monte Carlo approximation with 200 simulation iterations (Figure 5h). A dual-criterion selection strategy was applied to the RSF-derived SHAP results: genes were required to appear among the top 5 features by mean absolute SHAP importance and to exceed the median SHAP interquartile range (IQR) across all genes, with the IQR criterion capturing sample-level consistency of contributions. The top-5 importance ranking yielded ST6GALNAC1, PXN, HSPH1, CDKN2A, and FADD, while the IQR stability criterion retained ITGA5, SERPINE1, SCG5, ST6GALNAC1, and HSPH1. The intersection of both criteria identified ST6GALNAC1 and HSPH1 as the two genes combining the highest global prognostic importance with the most consistent per-sample contributions, designating them as priority targets for downstream characterisation. Threshold-sensitivity analysis showed that this pair was stable across all nine threshold combinations tested (top-K ∈ {3, 5, and 10}; IQR cut-off ∈ {none, median, and 75th percentile}) (Table S3).

### 3.5. Reciprocal Expression Patterns and Contrasting Immune Microenvironment Associations of HSPH1 and ST6GALNAC1

**Figure 6:**
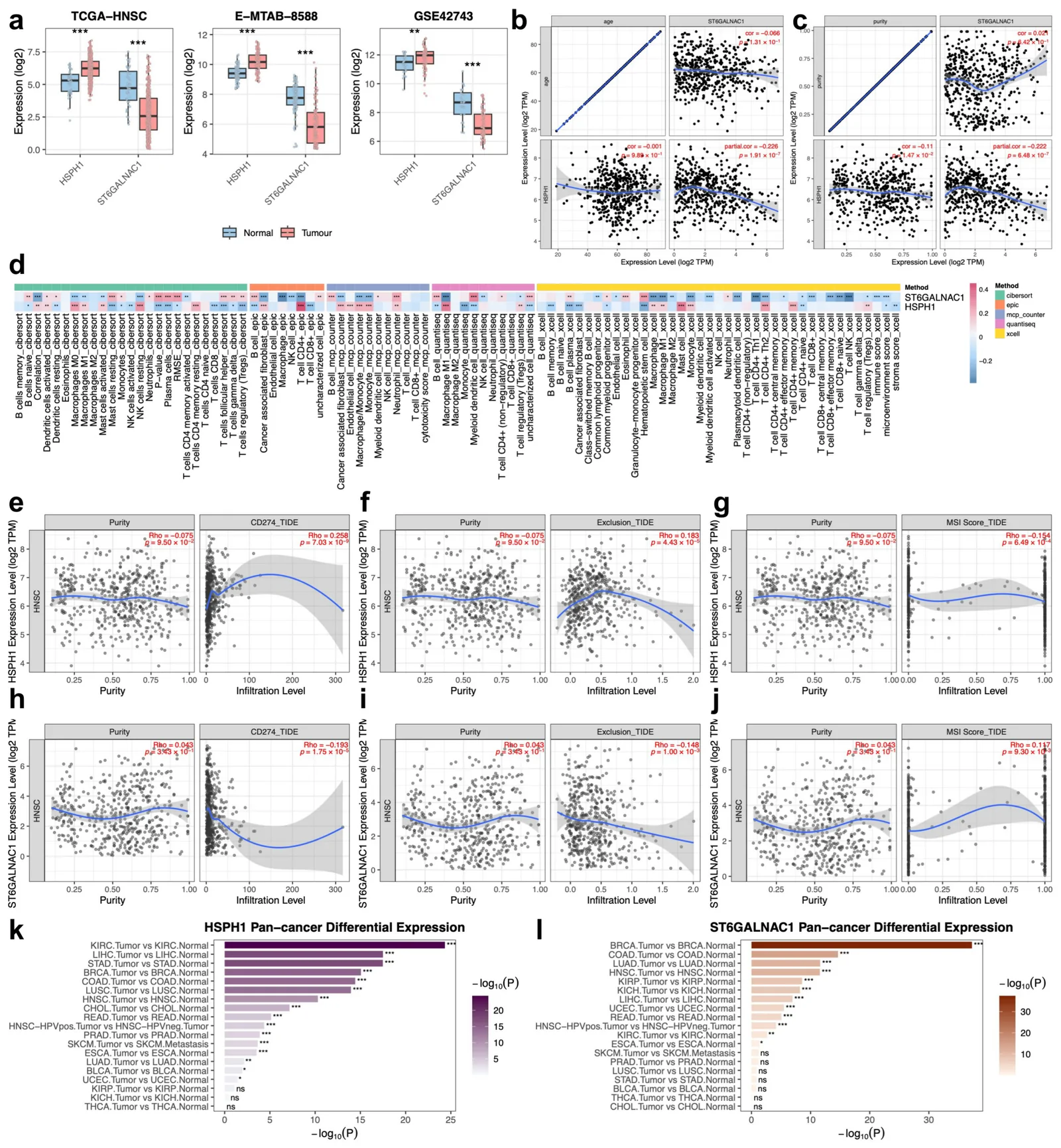
Reciprocal expression and contrasting tumour microenvironment associations of HSPH1 and ST6GALNAC1. (**a**) Expression of HSPH1 and ST6GALNAC1 in tumour vs. normal tissue in TCGA-HNSC, E-MTAB-8588 and GSE42743 (Wilcoxon rank-sum test): HSPH1 was upregulated and ST6GALNAC1 downregulated in tumour across all three cohorts. (**b**) Partial correlation between HSPH1 and ST6GALNAC1 expression after adjusting for age (partial r = −0.226, *p* = 1.91 × 10^−7^). (**c**) Partial correlation after adjusting for tumour purity (partial r = −0.222, *p* = 6.48 × 10^−7^). (**d**) Heatmap of Spearman correlations between HSPH1/ST6GALNAC1 expression and immune-cell fractions estimated by six deconvolution algorithms (TIMER, CIBERSORT, xCell, MCP-counter, EPIC and QUANTISEQ; columns grouped by method, significance indicated). (**e**–**g**) Correlations of HSPH1 expression with CD274 (PD-L1; Spearman rho = 0.258, adjusted *p* = 7.03 × 10^−9^), immune exclusion score (rho = 0.183, adjusted *p* = 4.43 × 10^−5^) and MSI score (rho = −0.154, adjusted *p* = 6.49 × 10^−4^); each panel also shows the non-significant correlation with tumour purity. (**h**–**j**) Corresponding correlations for ST6GALNAC1 with CD274 (rho = −0.193, adjusted *p* = 1.75 × 10^−5^), immune exclusion score (rho = −0.148, adjusted *p* = 1.00 × 10^−3^) and MSI score (rho = 0.117, adjusted *p* = 9.30 × 10^−3^). All *p*-values were Benjamini–Hochberg-adjusted across the tested correlation panel. (**k**) Pan-cancer differential expression of HSPH1 (16 tumour-vs.-normal comparisons; significant in 14 solid tumour types including KIRC, LIHC, STAD, BRCA, COAD, LUSC, CHOL, READ, PRAD, SKCM, ESCA, LUAD, BLCA and UCEC). (**l**) Pan-cancer differential expression of ST6GALNAC1 (significant in 10 tumour types including BRCA, COAD, LUAD, KIRP, KICH, LIHC, UCEC, READ, KIRC and ESCA). Asterisks indicate statistical significance: * *p* < 0.05; ** *p* < 0.01; *** *p* < 0.001.

SHAP analysis designated HSPH1 and ST6GALNAC1 as the two principal molecular contributors to SNI. To characterise their expression and immune landscape, we validated differential expression across the following three expression cohorts: TCGA-HNSC, E-MTAB-8588 (n = 215), and GSE42743. HSPH1 was consistently upregulated in tumour relative to normal tissue in all three datasets, while ST6GALNAC1 was consistently downregulated (Figure 6a). This reciprocal pattern accords with their opposing HRs (HSPH1 HR > 1; ST6GALNAC1 HR < 1) and their opposing SHAP sign contributions to SNI. Neither gene showed a significant correlation with patient age. After age adjustment, the two genes exhibited a significant inverse partial correlation (partial r = −0.226, *p* = 1.91 × 10^−7^; Figure 6b). After adjustment for tumour purity, the inverse relationship persisted (partial r = −0.222, *p* = 6.48 × 10^−7^; Figure 6c). HSPH1 expression was itself negatively correlated with tumour purity, suggesting that elevated HSPH1 is partly associated with the stromal and immune composition of the tumour microenvironment (TME), while ST6GALNAC1 showed no significant purity dependence.

TME deconvolution using six complementary algorithms (TIMER, CIBERSORT, xCell, MCP-counter, EPIC, and QUANTISEQ) applied to TCGA-HNSC bulk RNA-seq data revealed markedly divergent immune cell correlations for the two genes (Figure 6d). HSPH1 expression was positively correlated with CD274 (PD-L1; Figure 6e) and with the immune exclusion score (Figure 6f) and negatively correlated with microsatellite instability (MSI) scores (Figure 6g). ST6GALNAC1 showed the opposite pattern: negative correlations with CD274 (Figure 6h) and exclusion scores (Figure 6i) and a positive correlation with MSI (Figure 6j). These reciprocal immune associations mirror the checkpoint landscape differences between the C1 and C2 sialylation subtypes identified in the molecular subtyping analysis above, reinforcing a model in which HSPH1 and ST6GALNAC1 are biological surrogates for contrasting immune microenvironmental states. Pan-cancer analysis further identified differential HSPH1 expression in 14 solid tumour types, including KIRC, LIHC, STAD, BRCA, COAD, LUSC, CHOL, READ, PRAD, SKCM, ESCA, LUAD, BLCA, and UCEC (Figure 6k), and differential ST6GALNAC1 expression in 10 tumour types including BRCA, COAD, LUAD, KIRP, KICH, LIHC, UCEC, READ, KIRC, and ESCA (Figure 6l), suggesting that the opposing immunological associations of these two genes extend beyond HNSCC.

### 3.6. Single-Cell Transcriptomics Identifies Cell-Type-Specific Expression and a Cooperative Downstream Gene Network

**Figure 7:**
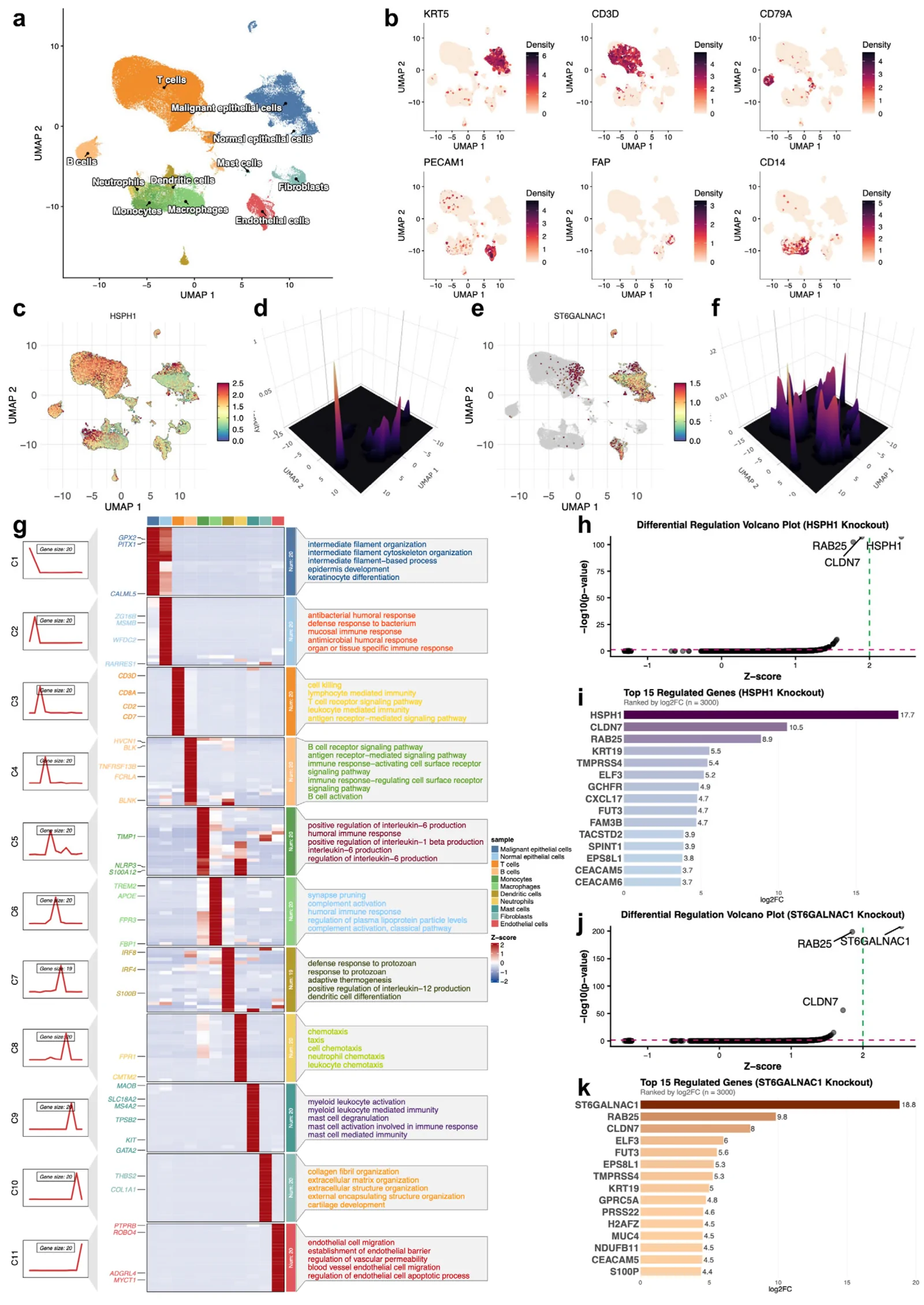
Single-cell transcriptomic dissection of HSPH1 and ST6GALNAC1 in the HNSCC microenvironment. (**a**) UMAP of 168,742 single cells from GSE234933 (52 HNSCC samples), coloured by cell type (malignant and normal epithelial cells, T cells, B cells, monocytes, macrophages, dendritic cells, neutrophils, mast cells, fibroblasts and endothelial cells). (**b**) UMAP density plots of canonical lineage markers (KRT5, CD3D, CD79A, PECAM1, FAP, CD14). (**c**) UMAP feature plot of HSPH1 expression. (**d**) Three-dimensional density surface of HSPH1 expression projected onto the UMAP embedding, showing highest density in malignant epithelial cells. (**e**) UMAP feature plot of ST6GALNAC1 expression. (**f**) Three-dimensional density surface of ST6GALNAC1 expression. (**g**) Cell-type-specific heatmap of marker-gene expression with GO biological-process enrichment terms for each cell type. (**h**) scTenifoldKnk virtual knockout of HSPH1 in tumour cells: volcano plot of differential regulation (Z-score vs. −log10 *p*-value), with CLDN7 and RAB25 among the most perturbed downstream genes. (**i**) Top 15 regulated genes after HSPH1 knockout ranked by log2FC (including CLDN7, RAB25, KRT19, ELF3, FUT3 and CEACAM5). (**j**) Volcano plot of differential regulation after ST6GALNAC1 knockout. (**k**) Top 15 regulated genes after ST6GALNAC1 knockout ranked by log2FC (including RAB25, CLDN7, ELF3, FUT3, KRT19 and CEACAM5).

To resolve the cell-type-specific contributions of HSPH1 and ST6GALNAC1 within the HNSCC microenvironment, we analysed single-cell RNA sequencing (scRNA-seq) data from the public dataset GSE234933 using Seurat-based preprocessing and canonical lineage marker-based cell-type annotation. Cells were assigned to major populations including malignant epithelial cells, normal epithelial cells, T cells, B cells, fibroblasts, and endothelial cells (Figure 7a,b). HSPH1 was most highly expressed in malignant epithelial cells, with secondary expression also present in T cells and B cells (Figure 7c,d). ST6GALNAC1 expression was predominantly concentrated in malignant cells, with lower-level expression observed in a subset of T cells and endothelial cells (Figure 7e,f). Cell-type-specific pathway enrichment analysis revealed that marker genes of malignant epithelial cells were enriched for intermediate filament organisation and cytoskeleton organisation, while T-cell markers were enriched for cell-killing and antigen-mediated signalling pathways (Figure 7g).

To predict transcriptional changes associated with in silico perturbation of each gene within the tumour compartment, we applied scTenifoldKnk to isolated tumour cell subpopulations. scTenifoldKnk is a computational virtual gene-knockout framework that uses single-cell network tensor decomposition and Euclidean distance-based differential expression to predict downstream transcriptional perturbation effects [37]. In silico knockout of HSPH1 in tumour cells produced the strongest downstream perturbations in CLDN7, RAB25, KRT19, ELF3, FUT3, and CEACAM5 (Figure 7h,i). Knockout of ST6GALNAC1 yielded a near-identical perturbation profile, with RAB25, CLDN7, ELF3, FUT3, KRT19, and CEACAM5 as the most affected downstream genes (Figure 7j,k). The high degree of overlap between the two perturbation networks is consistent with a potentially shared, computationally inferred regulatory network, with both genes predicted to affect overlapping components of a putative transcriptional programme in malignant cells. The shared effector genes span epithelial junction integrity (CLDN7), vesicle trafficking (RAB25), cytoskeletal architecture (KRT19), epithelial transcriptional control (ELF3), fucosyltransferase-mediated glycan modification (FUT3), and carcinoembryonic antigen-related immune modulation (CEACAM5), suggesting that the predicted effects of both genes overlap within a putative sialylation- and fucosylation-related transcriptional programme within the HNSCC tumour niche.

### 3.7. Spatial Transcriptomics Resolves the Tissue Architecture of the Sialylation Niche in HNSCC

**Figure 8:**
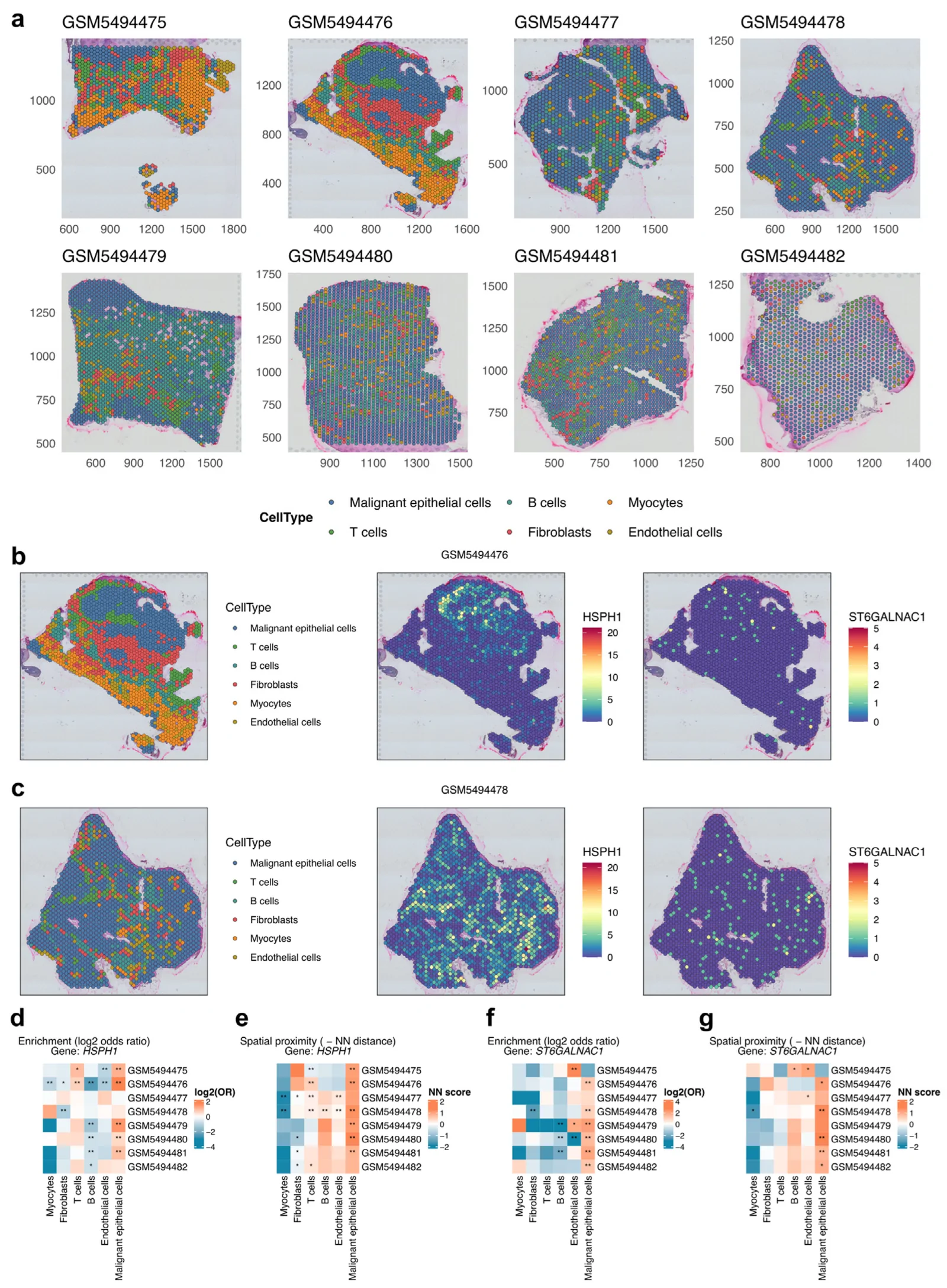
Spatial transcriptomics maps the sialylation niche within intact HNSCC tissue. (**a**) Cell-type-annotated spatial maps of the eight 10× Visium HNSCC sections from GSE181300 (malignant epithelial cells, T cells, B cells, endothelial cells, fibroblasts and myocytes), showing substantial inter-sample heterogeneity in cell-type composition. (**b**) Representative section GSM5494476 showing cell-type annotation together with HSPH1 and ST6GALNAC1 spatial expression; HSPH1-high spots co-localised with tumour and T-cell zones, whereas ST6GALNAC1-high spots were concentrated primarily in tumour cell regions. (**c**) Representative section GSM5494478 with the same layout. (**d**) Heatmap of HSPH1 cell-type enrichment (log2 odds ratio from Fisher’s exact test) across the eight sections. (**e**) Heatmap of HSPH1 nearest-neighbour spatial proximity scores (scaled −NN distance; permutation test, k = 6, 500 permutations) across the eight sections. (**f**,**g**) The corresponding enrichment and nearest-neighbour heatmaps for ST6GALNAC1, showing consistent association with tumour and endothelial cell zones. Asterisks indicate statistical significance: * *p* < 0.05; ** *p* < 0.01.

To map HSPH1 and ST6GALNAC1 expression within intact tumour tissue, we analysed spatial transcriptomics data from the GSE181300 dataset, comprising 10× Visium profiles from eight HNSCC tissue sections. Spots were annotated based on canonical marker genes to malignant epithelial cells, T cells, B cells, fibroblasts, endothelial cells, and myocytes. Substantial inter-sample heterogeneity in cell-type composition was evident across the eight specimens (Figure 8a), consistent with the known microenvironmental diversity of HNSCC.

Spatial expression mapping showed distinct distribution patterns for the two genes. HSPH1-high spots were enriched in regions populated by malignant epithelial and immune cells. In representative samples GSM5494476 (Figure 8b) and GSM5494478 (Figure 8c), HSPH1 signal was co-localised with annotated tumour and T-cell zones. ST6GALNAC1-high spots were distributed more broadly but were concentrated primarily in tumour cell regions, with secondary enrichment in endothelial cell areas. To quantify spatial associations objectively, we computed the following two complementary metrics: nearest-neighbour (NN) spatial significance, which tests whether gene-expressing spots cluster closer to a given cell type than expected by chance, and enrichment odds ratio (OR), which measures the relative overrepresentation of gene expression within each cell-type zone. Across the majority of samples, HSPH1 showed significant spatial co-enrichment with tumour cells, T cells, and B cells (Figure 8d,e), while ST6GALNAC1 spatial enrichment was most consistently associated with tumour cells and endothelial cells (Figure 8f,g). These tissue-level spatial findings were consistent with the single-cell expression profiles described above and supported the localisation of both HSPH1 and ST6GALNAC1 within the microenvironmental niche defined by spatial proximity to malignant cells and the surrounding stromal compartment in primary HNSCC tissue.

### 3.8. AI-Guided Multi-Stage Computational Screening Identifies Candidate Therapeutic Compounds Against HSPH1 and ST6GALNAC1

**Figure 9:**
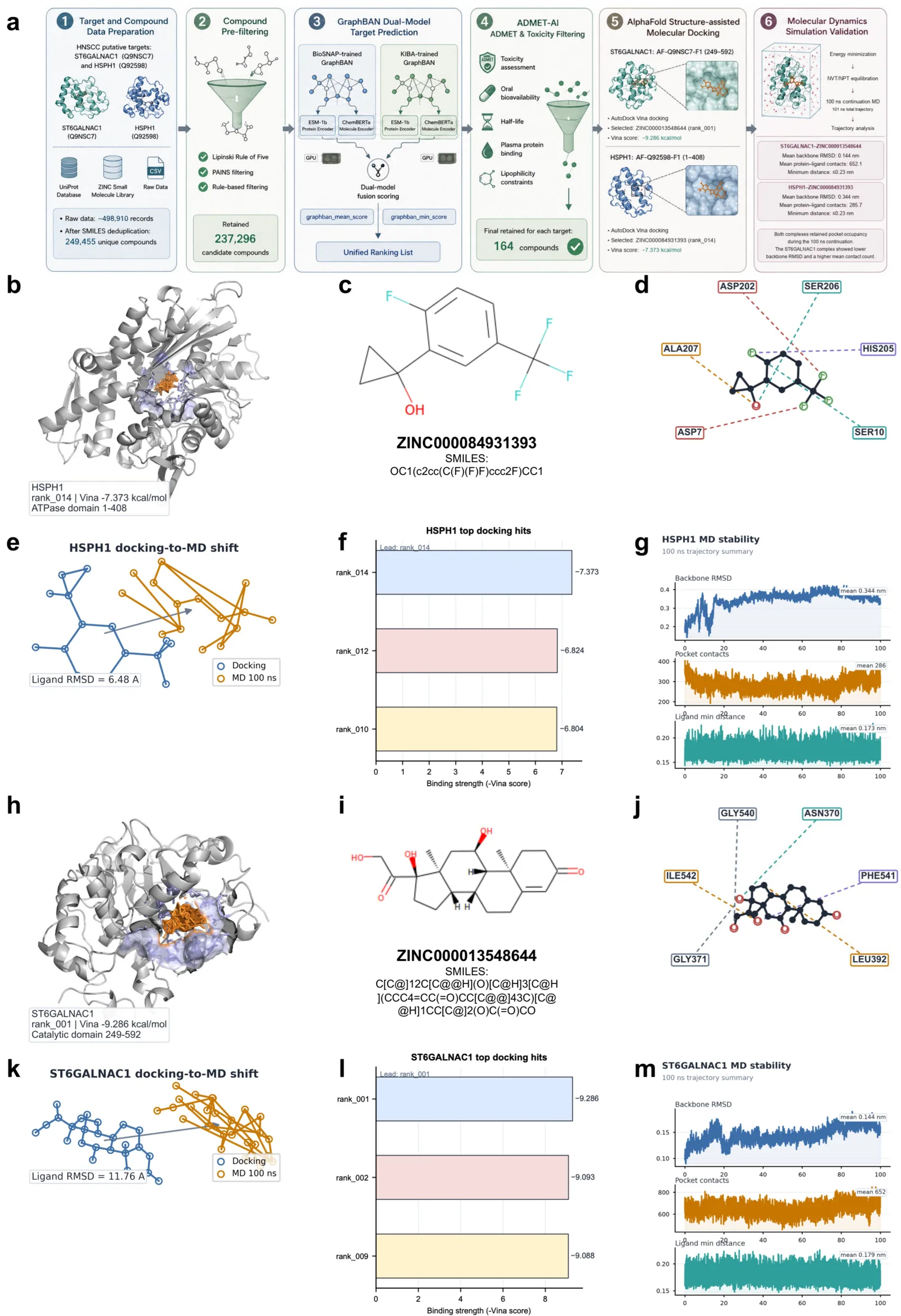
AI-guided multi-stage computational screening identifies candidate therapeutic compounds for HSPH1 and ST6GALNAC1. (**a**) Workflow of the target-centred computational screening: 249,455 unique ZINC compounds filtered by Lipinski’s rule of five and PAINS criteria, scored by dual GraphBAN models (BioSNAP and KIBA), filtered by ADMET-AI (164 candidates retained per target), re-ranked by AutoDock Vina docking of the top-20 candidates, followed by molecular dynamics assessment of the top-ranked complexes. (**b**) Three-dimensional binding mode of the top HSPH1 candidate, ZINC000084931393, docked into the nucleotide-binding cleft of the AlphaFold2-predicted HSPH1 ATPase/nucleotide-binding domain (pocket transferred from PDB 3FE1). (**c**) Two-dimensional chemical structure of ZINC000084931393 (SMILES shown). (**d**) Local interaction environment of the HSPH1 docked pose, showing residues within approximately 4.2 Å of the ligand. (**e**) Overlay of the original docked pose and the aligned 100 ns molecular dynamics (MD) endpoint of the HSPH1–ZINC000084931393 complex. (**f**) Docking-score ranking of the ADMET-prioritised candidates for HSPH1; ZINC000084931393 ranked first (−7.373 kcal/mol), followed by −6.824 and −6.804 kcal/mol. (**g**) Time-resolved MD trajectories of the HSPH1 complex (100 ns): backbone RMSD (mean 0.344 nm), protein–ligand contact counts (mean 285.7) and minimum protein–ligand distance. (**h**) Three-dimensional binding mode of the top ST6GALNAC1 candidate, ZINC000013548644, within the catalytic pocket of the AlphaFold2-predicted ST6GALNAC1 catalytic domain (pocket transferred from PDB 6APL). (**i**) Two-dimensional chemical structure of ZINC000013548644 (SMILES shown). (**j**) Local interaction environment of the ST6GALNAC1 docked pose (residues within approximately 4.2 Å). (**k**) Overlay of the docked pose and the aligned 100 ns MD endpoint of the ST6GALNAC1–ZINC000013548644 complex. (**l**) Docking-score ranking for ST6GALNAC1; ZINC000013548644 ranked first (−9.286 kcal/mol), followed by −9.093 and −9.088 kcal/mol. (**m**) Time-resolved MD trajectories of the ST6GALNAC1 complex (100 ns): backbone RMSD (mean 0.144 nm), protein–ligand contact counts (mean 652.1) and minimum protein–ligand distance.

Having established HSPH1 and ST6GALNAC1 as the principal molecular contributors to SNI, we implemented an AI-guided multi-stage computational pipeline to identify small-molecule candidates targeting these two proteins. The overall workflow is summarised in Figure 9a. Starting from a ZINC-scale library of 249,455 unique compounds, compound–target interaction prediction was performed using GraphBAN [38], a graph-based bilinear attention network that jointly encodes drug molecular graphs and protein sequences to predict binding probability. Two independently trained GraphBAN models derived from the BioSNAP and KIBA interaction datasets were applied in parallel to reduce single-model bias. Pharmacokinetic and toxicological profiling was then carried out using ADMET-AI (absorption, distribution, metabolism, excretion, and toxicity-AI) [39], a deep-learning ADMET prediction framework. Multi-criteria filtering retained 164 drug-like candidates per target, representing a set that simultaneously satisfies model-based interaction priority and predicted criteria related to oral bioavailability, metabolic stability, and toxicity risk.

For HSPH1, structure-based docking was performed against the AlphaFold-predicted ATPase/nucleotide-binding domain of HSPH1, with the binding pocket transferred from the ADP-bound homologous structure. The three-dimensional binding mode of the top candidate, ZINC000084931393, is shown in Figure 9b, where the docked pose occupies the nucleotide-binding cleft of the receptor. The two-dimensional chemical structure of ZINC000084931393 is presented in Figure 9c. The local interaction environment around the docked pose, showing residues within approximately 4.2 Å, of the ligand is depicted in Figure 9d. Among all ADMET-prioritised candidates advanced to docking, ZINC000084931393 achieved the most favourable docking score (−7.373 kcal/mol), followed by the second- and third-ranked compounds (−6.824 kcal/mol and −6.804 kcal/mol, respectively; Figure 9f). Notably, ZINC000084931393 emerged as the top-ranked structural candidate through docking-based re-ranking despite not occupying the top position in the preceding ADMET stage, illustrating the added discriminatory value of integrating three-dimensional pocket geometry into the prioritisation.

To characterise the trajectory stability of the HSPH1-ZINC000084931393 complex, we performed a 100 ns molecular dynamics (MD) simulation. The overlay of the original docked pose with the aligned 100 ns MD endpoint (Figure 9e) shows continued ligand occupancy of the nucleotide-binding cleft over the simulation window. Time-resolved traces of backbone root-mean-square deviation (RMSD), protein–ligand contact counts, and minimum protein–ligand distance are shown in Figure 9g. The mean backbone RMSD was 0.344 nm, and the mean protein-ligand contact count was 285.7. The RMSD rose to a plateau of 0.36–0.38 nm within the first 40 ns, a relaxation attributable mainly to the flexible C-terminal region (residues 404–408, with RMSF up to 1.0 nm) rather than to the binding site, which retained the ligand throughout (minimum protein–ligand distance never exceeded 0.23 nm).

For ST6GALNAC1, docking was performed against the AlphaFold-predicted catalytic domain, with the binding pocket transferred from a homologous human sialyltransferase structure. The three-dimensional binding mode of the top candidate, ZINC000013548644, positioned within the transferred catalytic pocket, is shown in Figure 9h. The two-dimensional chemical structure of ZINC000013548644 is presented in Figure 9i. The local interaction environment around the docked pose, showing nearby residues within approximately 4.2 Å of the ligand conformation, is depicted in Figure 9j. ZINC000013548644 achieved the most favourable docking score among the tested candidates (−9.286 kcal/mol), with the second- and third-ranked compounds reaching −9.093 kcal/mol and −9.088 kcal/mol, respectively (Figure 9l). The concordance of ZINC000013548644’s top ranking across the GraphBAN scoring, ADMET filtering, and docking stages supports the internal consistency of the multi-stage prioritisation for this target.

The 100 ns MD simulation of the ST6GALNAC1-ZINC000013548644 complex showed compact structural behaviour throughout the trajectory. The overlay of the docked pose and the 100 ns MD endpoint (Figure 9k) revealed continued ligand occupancy of the transferred pocket over the simulation window (minimum protein–ligand distance never exceeded 0.23 nm). Time-resolved MD traces (Figure 9m) showed a mean backbone RMSD of 0.144 nm and a mean protein–ligand contact count of 652.1, indicating greater trajectory stability over the simulated window relative to the HSPH1 complex. In both complexes, the interactions observed during the trajectory included hydrophobic packing, van der Waals complementarity, and intermittent hydrogen bonds (mean 0.97 and 0.80 per frame in the HSPH1 and ST6GALNAC1 complexes, respectively). Taken together, the multi-stage AI-guided pipeline identified ZINC000013548644 and ZINC000084931393 as putative candidates for ST6GALNAC1 and HSPH1, respectively, providing a structurally supported and pharmacologically filtered starting point for further pharmacological investigation of the sialylation niche in HNSCC.

Across the full screening cascade, both putative candidates received concordant computational prioritisation across multiple complementary evaluation stages as follows: model-based interaction prediction, multi-criteria ADMET filtering, structure-based docking, and 100 ns trajectory behaviour. A direct comparison of the two complexes highlights distinct computational trajectory profiles. ZINC000013548644 targeting ST6GALNAC1 showed a lower backbone RMSD over the simulated window (0.144 nm versus 0.344 nm) and a higher mean protein-ligand contact count (652.1 versus 285.7) relative to the HSPH1 complex, as well as a more favourable docking score (−9.286 kcal/mol versus −7.373 kcal/mol). Both compounds met the same predefined ADMET filtering criteria, although their individual predicted pharmacokinetic and toxicity profiles require separate evaluation. From a biological perspective, HSPH1 and ST6GALNAC1 show opposing associations with HNSCC prognosis and immune microenvironmental state, with HSPH1 upregulation associated with an immunosuppressed, PD-L1-high phenotype and ST6GALNAC1 downregulation linked to reduced sialylation; higher ST6GALNAC1 expression was weakly associated with higher microsatellite instability scores. The identification of structurally distinct small-molecule candidates against each target therefore offers a dual-axis approach to modulating the sialylation niche: suppressing HSPH1-associated immune exclusion while modulating ST6GALNAC1-associated glycan signalling. These computational findings establish a prioritised compound set and a conceptual framework for subsequent cell-based and in vivo evaluation in HNSCC.

To provide indirect target-level context from public pharmacogenomic data, we profiled the expression–drug-sensitivity associations of the two target genes across three public cell-line panels (GDSC2, CTRP, and DepMap PRISM) and an oncoPredict bridge to TCGA-HNSC (Figures S4–S7; Table S4a–d). High ST6GALNAC1 expression marked sensitivity to MEK/ERK-pathway inhibitors in both the GDSC2 and CTRP panels—the most robust cross-panel signal—while higher HSPH1 expression marked sensitivity to WEE1/CHK1 and BCL-2-family inhibitors in GDSC2. Effect sizes were modest, and the HNSCC-restricted GDSC2 subset (n = 29) yielded no significant associations, consistent with limited statistical power.

## 4. Discussion

Sialylation remodels the tumour glycocalyx and suppresses anti-tumour immunity, yet its prognostic landscape in HNSCC has not been systematically mapped [14]. We addressed this by intersecting 1204 curated sialylation genes with transcriptomic data from seven GEO cohorts (n = 476) and TCGA-HNSC (n = 501) and then applying a multi-layer analytical framework as follows: consensus clustering, benchmarking of 117 machine-learning algorithm combinations (nominally 101), SHAP feature attribution, single-cell transcriptomics, spatial mapping, and AI-guided drug discovery. The resulting SNI distils the sialylation axis into a prognostic score whose performance is reported cohort by cohort: C-index 0.88 in the training cohort (bootstrap 95% CI 0.860–0.894), 0.68 in the external model-selection cohort GSE42743, and 0.58–0.62 in the three independent external test cohorts, indicating retained but attenuated prognostic signal upon cross-platform transfer. SHAP analysis identified the following two principal contributors with opposing associations: HSPH1 is upregulated in HNSCC and confers excess risk (HR 1.24), whereas ST6GALNAC1 is downregulated and is protective (HR 0.88). Despite these opposing associations, both genes converge on the same downstream regulatory network. This computationally inferred convergence is the central mechanistic hypothesis of this work, and it structures much of the discussion below.

The two-cluster subtyping partitioned TCGA-HNSC patients into a sialylation-enriched C1 group and an immune-active C2 group. The immune landscape of C1 is not simply PD-L1-high. TIM-3, PD-L2, and SIGLEC15 are co-elevated, indicating that multiple inhibitory circuits are simultaneously active. Doescher et al. showed that co-expression of PD-L1, TIM-3, and LAG-3 characterised non-responding HNSCC tumours after chemoradiotherapy [40]. Our data extend this picture to include Siglec-class suppression. Wang et al. established that SIGLEC15 suppresses T-cell proliferation through a mechanism independent of and often mutually exclusive with PD-L1 [34]. Its co-elevation with PD-L1 in C1 implies that blocking PD-1 alone leaves a parallel inhibitory circuit intact. The GSEA enrichment of C1 included focal adhesion, ECM–receptor interaction, and integrin signalling. These signatures point to an immune-excluded rather than immune-desert phenotype: stromal remodelling impedes T-cell penetration without eliminating immune cells from the tissue altogether. Clinically, this profile suggests that C1 patients may require combination strategies that address both Siglec–sialic axis suppression and stromal exclusion.

The SNI was selected from 117 machine-learning algorithm combinations (nominally 101) according to the highest mean C-index across the training cohort and the external model-selection cohort GSE42743; the three external cohorts never entered model selection. This design reduces model selection bias, although optimism arising from screening 117 variants remains possible. The hybrid StepCox[forward] + RSF architecture captures linear hazard associations in the variable selection step and non-linear survival dependencies in the forest component. In TCGA-HNSC the C-index was 0.88 (bootstrap 95% CI 0.860–0.894); in GSE42743 it was 0.68, and in the three independent external test cohorts 0.62 (E-MTAB-8588), 0.58 (GSE65858), and 0.62 (GSE41613). Alabi et al. reported C-indices in the 0.72–0.80 range for HNSCC explainable survival models [41]. SNI performance is therefore competitive while being anchored to a defined biological axis [14]; the complete 117-variant benchmark of the Mime1 framework [42] is provided in Table S2. In multivariable Cox regression, SNI was independent of conventional staging categories (HR 5.49, *p* < 0.001). Age was the only clinical variable that remained co-significant (HR 1.02). The age association is biologically coherent given the relationship between immunosenescence and tumour immune evasion. The nomogram incorporating age and SNI achieved AUC values of 0.918, 0.943, and 0.955 at one, two, and three years, respectively, providing a clinically interpretable format for future prospective evaluation.

The performance gradient across cohorts merits explicit interpretation. GSE42743, the external model-selection cohort, comprises HPV-negative oral squamous cell carcinoma profiled on the same Affymetrix platform (GPL570) as most of the discovery cohorts and is a widely used external cohort in HNSCC prognostic-signature studies [25]; its clinical composition is closest to the predominantly HPV-negative TCGA-HNSC training population. The following three independent external test cohorts differ in ways that plausibly attenuate transferability: E-MTAB-8588 mixes HPV-positive and HPV-negative tumours profiled on Illumina HumanHT-12 arrays; GSE65858 is an oral-cavity-only cohort on GPL10558 arrays, a platform on which no model in the benchmark exceeded a C-index of 0.61; and GSE41613, although HPV-negative, records follow-up in months and originates from the same study as GSE42743 [25]. Differences in platform, HPV composition, anatomical mix, follow-up conventions, and treatment era can all shift a score’s discrimination, and a signature trained on one platform is not expected to reach identical C-indices on another without recalibration. Accordingly, we interpret the external results as follows: the SNI retains statistically significant, directionally consistent prognostic signal in every external cohort (pooled stratified HR 1.016 per unit risk score, 95% CI 1.010–1.021, *p* = 1.95 × 10^−7^, and n = 524), while the moderate per-cohort C-indices (0.58–0.62) indicate that direct clinical application to new populations should be preceded by recalibration. Prospective validation in independent clinical cohorts with standardised sample collection and annotation is needed to establish calibration and generalisability across institutions and treatment settings.

HSPH1 encodes HSP110, the largest member of the HSP70 superfamily. It functions as a nucleotide exchange factor and disaggregation co-chaperone, helping cells manage proteotoxic stress. Fan et al. showed that HSPH1 is upregulated in HNSCC relative to normal mucosa and predicts inferior overall survival, consistent with our three-cohort validation [43]. Yan et al. observed similar dysregulation across 33 TCGA tumour types, pointing to a broad oncogenic role [44]. The molecular basis may involve STAT3. Berthenet et al. demonstrated that HSP110 drives STAT3 phosphorylation and nuclear translocation in colorectal cancer [45]. STAT3 activation is itself a well-established driver of PD-L1 transcription and immune suppression. If this axis is operative in HNSCC, HSPH1 overexpression would simultaneously maintain tumour cell survival and sustain an immunosuppressive signalling state. The positive correlation of HSPH1 with PD-L1 expression and the immune exclusion score in our data is consistent with this model. Wu et al. further showed that HSPH1 is released in extracellular vesicles and influences CD8+ T-cell stress responses through the IL-2/MYC/IL-2RA pathway [46]. The spatial data add an important nuance. HSPH1-high spots co-localise with annotated T-cell zones, yet these same regions display high immune exclusion scores. Immune cells are present; they are not functioning. We propose that HSPH1 creates a state of immune dysfunction rather than immune absence, analogous to a functional checkpoint imposed at the microenvironmental level. Pharmacological inhibition of HSPH1 could, in principle, disrupt this proteostatic–immunosuppressive coupling and restore T-cell effector function. HSPH1 is considerably less explored as a drug target than HSP90 or HSP70, and its ATPase/nucleotide-binding domain offers structural selectivity that distinguishes it from other family members.

ST6GALNAC1 tells a contrasting story. It encodes an alpha-2,6-sialyltransferase that transfers sialic acid to O-GalNAc residues, producing sialyl-Tn (sTn) antigen on mucins and surface glycoproteins. In HNSCC, ST6GALNAC1 is consistently downregulated in tumour relative to normal mucosa across all three expression cohorts; in TCGA-HNSC, higher expression was associated with better survival (univariate Cox regression; HR 0.88, 95% CI 0.81–0.96, and *p* = 0.006). This protective effect contrasts with the oncogenic role of ST6GALNAC1 in other epithelial malignancies. In ovarian cancer, ST6GALNAC1 is highly expressed in cancer stem cells and promotes proliferation, migration, and self-renewal through Akt-dependent signalling [47]. Conversely, in cholangiocarcinoma, loss of core 1 O-glycosylation is accompanied by ST6GALNAC1 downregulation and concurrent AKT/ERK activation [48], illustrating that ST6GALNAC1 dysregulation is a recurrent event across tumour types, though with divergent directional effects. The discrepancy is likely context-dependent. In lung cancer, ST6GALNAC1 upregulation promotes immune evasion through sialylation of NECTIN2 and MUC5AC [49,50]. In normal HNSCC mucosa, ST6GALNAC1 may instead maintain a glycan programme that supports ordinary epithelial identity. Its loss in tumour cells could disrupt this programme, shifting the glycocalyx toward sialylation patterns that more potently engage inhibitory Siglec receptors on myeloid and NK cells [51,52]. Under this model, ST6GALNAC1 downregulation is not merely a passenger event; it may actively remodel the sialylation niche toward immune suppression. The positive correlation of ST6GALNAC1 with MSI scores is consistent with this interpretation. Microsatellite-unstable tumours carry greater neoantigen burdens and tend toward more permissive immune environments, and they appear to retain higher ST6GALNAC1 expression. Whether ST6GALNAC1 loss precedes or follows immune exclusion remains to be determined. Nevertheless, its inverse correlation with PD-L1 and its pan-cancer differential expression across 10 TCGA tumour types (Figure 6l) suggest that restoring ST6GALNAC1-dependent glycan signalling could represent a therapeutically meaningful intervention. The goal would be to normalise the glycocalyx rather than to add another checkpoint blockade agent.

That two genes with opposing prognostic associations share a common downstream perturbation profile is, at first reading, paradoxical. In silico knockout of HSPH1 or ST6GALNAC1 in tumour cells produced near-identical transcriptional effects, with CLDN7, RAB25, KRT19, ELF3, FUT3, and CEACAM5 as the principal effectors in both cases. A plausible interpretation is that the predicted effects of HSPH1 and ST6GALNAC1 overlap on a shared glycan–cytoskeletal axis in malignant epithelial cells, and that virtual perturbation of either gene is predicted to affect the same downstream programme. CLDN7 encodes a tight junction claudin; its disruption is associated with epithelial–mesenchymal transition and invasion [53], though pro-tumorigenic roles have also been reported in specific contexts [54]. RAB25 regulates endosomal recycling and integrin trafficking; its cancer role is bidirectional depending on tumour. ELF3 is an epithelial-specific ETS transcription factor that maintains epithelial identity and actively represses EMT [55,56]. FUT3-mediated fucosylation competes with sialylation for the same glycan substrates, and Lewis antigen expression driven by FUT3 has been linked to tumour progression and chemotherapy resistance [57]. CEACAM5 modulates anti-tumour immunity and correlates with stemness in solid tumours [58,59]. The convergence of these effectors suggests that the predicted effects of HSPH1 and ST6GALNAC1 may be associated with overlapping components of the epithelial glycan–junction architecture of the HNSCC niche, notwithstanding their opposing prognostic associations in the clinical transcriptome. Virtual perturbation of either gene produced overlapping predicted downstream changes.

Bulk RNA-seq provided the foundation for SNI and the clinical subtyping, but it cannot resolve the cell types responsible for the observed signals. Single-cell RNA sequencing of 168,742 cells from 52 HNSCC samples filled this gap. HSPH1 was most highly expressed in malignant epithelial cells, with secondary expression in T cells and B cells. This dual distribution is consistent with HSPH1’s proposed role both as a tumour-intrinsic survival factor and as an extracellular vesicle cargo influencing immune cell responses [46]. ST6GALNAC1 was concentrated in malignant and endothelial cells. The endothelial signal is of interest given the established role of ST6GALNAC1 in angiogenesis regulation [50], raising the possibility that sialylation of vascular endothelium contributes to the structural organisation of the sialylation niche. The spatial transcriptomics data from eight 10× Visium sections corroborated these assignments. HSPH1-high spots co-localised with tumour and T-cell zones; ST6GALNAC1-high spots were primarily tumour-associated with secondary endothelial enrichment. These spatial patterns match what prior HNSCC spatial studies have reported: tumour-core and invasive-edge transcriptional architectures are conserved across patients [18], cancer-associated fibroblasts restrict CD8+ T-cell infiltration [15], and ecosystem trajectories are structured across epithelial, stromal, and immune compartments [19]. Our data add a new layer to this picture: the sialylation gene programme maps onto a spatially coherent tissue domain within these previously described architectures. Glycomic profiling of HNSCC cell lines has shown that 24–35% of N-glycans carry sialylated modifications, with HPV status modulating the overall sialylation profile [60]. Sialylation is therefore a pervasive feature of the HNSCC tissue environment, not a marginal one.

The AI-guided drug discovery pipeline screened 249,455 ZINC compounds through the following four sequential filters: GraphBAN-based drug–target interaction prediction [38], ADMET-AI pharmacological profiling [39], AlphaFold2 structure-based docking [61,62], and 100 ns molecular dynamics simulation. The multi-stage design is intentional. Each filter eliminates different failure modes as follows: graph neural network models such as GraphBAN [38] encode protein sequence context that ligand-based screens lack, and related graph-based frameworks predict anticancer drug-combination synergy from heterogeneous biological networks [63]; ADMET filtering removes compounds with poor drug-likeness before docking; AlphaFold2-predicted structures are increasingly validated for virtual screening applications [61,62]. For HSPH1, ZINC000084931393 was docked into the ATPase/nucleotide-binding cleft with a docking score of −7.373 kcal/mol. Mean backbone RMSD over the 100 ns trajectory was 0.344 nm with 285.7 protein–ligand contacts; the RMSD rise reflects relaxation of the flexible C-terminal region rather than of the binding site, which retained the ligand throughout. The therapeutic hypothesis is direct: if HSPH1 promotes immune exclusion through the STAT3-PD-L1 axis, a compound occupying the nucleotide-binding cleft could disrupt this cascade at its upstream source. For ST6GALNAC1, ZINC000013548644 was docked into the catalytic domain with a docking score of −9.286 kcal/mol. The mean RMSD was 0.144 nm and the mean contact count was 652.1, indicating a more compact interface and greater trajectory stability. The therapeutic rationale here is more nuanced. Because ST6GALNAC1 is a protective gene that is already downregulated in tumour cells, the functional outcome of ZINC000013548644 binding depends on whether the compound modulates enzyme activity in an agonistic or antagonistic direction. If the binding conformation promotes ST6GALNAC1 catalytic activity, it may help restore a normal glycan landscape in tumour cells, potentially reducing alternative Siglec-mediated immune suppression. Experimental characterisation of the compound’s functional mode is a prerequisite for advancing this hypothesis. Together, the two compounds represent dual-axis target-engagement candidates requiring biochemical determination of their functional modes: one agent targeting HSPH1-associated immune exclusion in tumour cells, another aimed at modulating ST6GALNAC1-dependent glycan signalling in the tumour niche.

AlphaFold-based structure prediction underpins the docking stage of this pipeline [64], and the field continues to evolve rapidly. A recent scientometric analysis of the AlphaFold research landscape documented an annual growth rate of approximately 180%, with hotspots concentrated in structure prediction, drug discovery, and molecular dynamics [65], while the utility of AlphaFold2 models as docking receptors for virtual screening has been assessed favourably in dedicated benchmarks [21,61,62]. In this study, the AlphaFold2 models of HSPH1 (AF-Q92598-F1) and ST6GALNAC1 (AF-Q9NSC7-F1) served as the docking receptors, with binding pockets transferred from homologous experimental structures (PDB 3FE1 and 6APL) to compensate for the absence of bound-ligand information in the predicted models. The limitations of this approach are acknowledged: AlphaFold structures are predictions rather than experimentally resolved co-complexes, side-chain conformations within transferred pockets can deviate from holo structures, and pocket transfer introduces template bias; docking scores and MD trajectories therefore rank and retain candidates rather than prove binding. These caveats motivate the experimental binding and activity assays proposed below.

Because the screening cascade is entirely computational, we contextualised the two targets using public pharmacogenomic resources. In the GDSC2 panel (805 cell lines, 198 drugs), higher HSPH1 expression was associated with sensitivity to WEE1/CHK1 checkpoint inhibitors and BCL-2-family inhibitors (|rho| ≈ 0.10–0.12), while higher ST6GALNAC1 expression was associated with sensitivity to MEK/ERK-pathway inhibitors (SCH772984 rho = −0.22; trametinib rho = −0.22; and selumetinib rho = −0.21). The ST6GALNAC1–MEK/ERK sensitivity pattern was replicated in a second pharmacogenomic resource (the CTRP panel) (829 cell lines, 545 drugs), where ST6GALNAC1-high lines were additionally sensitive to EGFR/ERBB-family inhibitors (afatinib rho = −0.30; erlotinib rho = −0.27) and resistant to ferroptosis inducers (ML162 rho = +0.30). This cross-panel replication is the most robust of the pharmacogenomic signals. In the DepMap PRISM panel (477 cell lines, 1448 drugs), HSPH1 reproduced an EGFR/ERBB-family sensitivity cluster (91 significant associations; afatinib rho = −0.28), whereas ST6GALNAC1 showed only three significant associations—effectively a null result, which we report in full: the strong ST6GALNAC1 signals seen in GDSC2/CTRP do not transfer to the PRISM panel. Effect sizes in these panel-wide correlations are modest, and significance is driven by panel size; the head-and-neck-restricted GDSC2 subset (n = 29) showed no significant associations for either gene, a power-limited null. To bridge toward patients, we transferred GDSC2 drug-response models onto TCGA-HNSC expression (n = 501) with oncoPredict, removing the two target genes from the feature space to avoid circularity; effect sizes are the honest readout here. High ST6GALNAC1 expression was associated with predicted resistance to proteasome inhibitors (MG-132 rho = +0.61; bortezomib rho = +0.44) and to the HSP90 inhibitor luminespib (rho = +0.57), and with predicted sensitivity to the aurora kinase inhibitor ZM447439 (rho = −0.46), with HSPH1 showing the mirror pattern for MG-132 (rho = −0.13). These TCGA-level associations are hypothesis-generating—no patient-level drug-response ground truth exists for TCGA—but they translate the panel-level pharmacogenomics into testable clinical hypotheses, notably that ST6GALNAC1-high tumours may be preferentially sensitive to MEK/ERK inhibition yet resistant to proteasome inhibition (Table S4a–d; Figures S4–S7).

Relative to the existing chaperone-inhibitor landscape, HSPH1 (HSP110) is considerably less explored than HSP90 and HSP70, and its ATPase/nucleotide-binding domain offers structural selectivity that distinguishes it from other family members; ZINC000084931393 therefore represents a structurally supported starting point rather than a repurposing of existing inhibitors. For ST6GALNAC1, a sialyltransferase with no approved inhibitor, whether ZINC000013548644 acts in an agonistic or antagonistic direction remains to be determined. A staged experimental programme follows directly from these results as follows: (i) expression-stratified viability profiling of MEK/ERK inhibitors in ST6GALNAC1-low versus -high HNSCC cell lines to test the GDSC2/CTRP-derived hypothesis; (ii) recombinant-enzyme and cellular binding assays (e.g., thermal shift and enzyme-activity readouts) for the two docked candidates; (iii) co-culture immune-killing assays and syngeneic models to test whether HSPH1 inhibition relieves immune exclusion; and (iv) prospective cohort studies to establish the clinical calibration of the SNI. These steps are the subject of the subsequent experimental phase of this programme; the present work defines the targets, the candidate compounds, and the prioritisation rationale.

This study integrates seven GEO cohorts, TCGA-HNSC, single-cell RNA sequencing of 168,742 cells, 10× Visium spatial transcriptomics from eight tumour sections, and an AI-guided drug discovery pipeline into a single analytical programme. Its principal strengths are the anchoring of a prognostic model to a defined biological axis rather than agnostic features, the full disclosure of per-cohort performance across five cohorts—including the complete benchmark of 117 model variants, which quantifies selection instability— and the coupling of single-cell and spatial characterisation with dual-target candidate prioritisation, which together provide a concrete path from glycan biology toward clinical and experimental translation.

This study is subject to four limitations. First, the SNI was derived and validated in retrospective bulk transcriptomic cohorts, and its cross-cohort C-indices indicate that transfer to new populations requires recalibration; prospective validation in independent clinical cohorts with standardised sample collection and clinical annotation is needed. Second, although the 100 ns molecular dynamics simulations supported the persistence of both complexes within their binding pockets, this timescale remains short relative to slow conformational changes and thermodynamic binding equilibria; free-energy perturbation calculations and further simulation are required before compound prioritisation can be fully justified. Third, the mechanistic conclusions and drug candidates derived here are computational predictions; cell-based assays and in vivo efficacy studies are the necessary experimental programme that follows from this work. Fourth, the spatial transcriptomic findings derive from eight tissue sections and are therefore discovery-level; population-level conclusions about the spatial niche require larger, multi-institution cohorts. This study is best understood as a hypothesis-generating platform: it defines the targets, identifies the candidate compounds, and provides structural and transcriptomic rationale for prioritisation, rather than claiming experimental proof of mechanism.

The natural next steps follow from this framing. For the model, multi-institution prospective cohorts and platform-specific recalibration are the priority. For the mechanism, functional perturbation studies—knockdown or rescue of HSPH1 and ST6GALNAC1 in HNSCC lines followed by glycocalyx profiling and immune co-culture readouts—would test the shared glycan-junction hypothesis directly. For the compounds, the binding and activity assays outlined above would determine whether either candidate modulates its target in the predicted direction. Free-energy perturbation calculations are also warranted to consolidate the binding-affinity claims. Each of these directions is experimentally defined and, collectively, they outline the route from the computational framework presented here to a validated translational programme.

## 5. Conclusions

This study establishes a comprehensive, sialylation-specific multi-omic framework for HNSCC. Starting from 156 sialylation-associated DEGs, 31 prognostically significant genes were identified; these genes define two immunologically distinct subtypes (C1 and C2) and underpin the SNI prognostic model, whose performance we report cohort by cohort (C-index 0.88 in TCGA-HNSC; 0.68 in the external model-selection cohort GSE42743; and 0.58–0.62 in three independent external test cohorts). SHAP analysis identified HSPH1, an upregulated risk gene that likely sustains immune exclusion through STAT3-PD-L1 signalling, and ST6GALNAC1, a protective gene whose downregulation in tumour cells may destabilise normal glycan–immune interactions, as the two principal contributors; both converge on a shared downstream network encompassing CLDN7, RAB25, ELF3, FUT3, and CEACAM5 and co-localise within the tumour niche at single-cell and spatial resolution. The AI-guided screening cascade nominated ZINC000084931393 and ZINC000013548644 as structurally supported putative candidates against HSPH1 and ST6GALNAC1, respectively. Sialylation thus represents a molecularly distinct immunosuppressive axis in HNSCC, separate from and potentially complementary to PD-1 blockade. Experimental validation of the candidate compounds, the proposed mechanisms, and the clinical utility of the SNI in prospective cohorts—with recalibration as indicated by the cross-cohort data—is the essential next step toward patient benefit.

## Figures and Tables

**Table 1:** Transcriptomic datasets used in this study. GSE41613 follow-up was recorded in months; its time-dependent AUCs were computed at 12, 24, and 36 months (equivalent to 1, 2, and 3 years).

Dataset	Platform	Samples (*n*)	Usage
GSE29330	GPL570 (Affymetrix U133 Plus 2.0)	18	DEG discovery (tumour vs. normal)
GSE30784	GPL570 (Affymetrix U133 Plus 2.0)	212	DEG discovery (tumour vs. normal)
GSE3292	GPL570 (Affymetrix U133 Plus 2.0)	36	DEG discovery (tumour vs. normal)
GSE42743	GPL570 (Affymetrix U133 Plus 2.0)	103 (74 with OS; 42 events)	DEG discovery (tumour vs. normal); external model selection (74 with OS)
GSE6791	GPL570 (Affymetrix U133 Plus 2.0)	56	DEG discovery (tumour vs. normal)
GSE7224	GPL570 (Affymetrix U133 Plus 2.0)	13	DEG discovery (tumour vs. normal)
GSE9844	GPL570 (Affymetrix U133 Plus 2.0)	38	DEG discovery (tumour vs. normal)
TCGA-HNSC	RNA-seq (Illumina HiSeq; no GPL ID)	501 (219 events)	Training: feature/model selection and SNI fitting
E-MTAB-8588	Illumina HumanHT-12 (ArrayExpress A-MEXP-1173)	215 (83 with OS; 53 events)	Independent external test
GSE65858	GPL10558 (Illumina HumanHT-12 v4)	270 (94 events)	Independent external test
GSE41613	GPL570 (Affymetrix U133 Plus 2.0)	97 (51 events; follow-up in months)	Independent external test

**Table 2:** Performance of the SNI model (StepCox[forward] + RSF) across the five scored cohorts. GSE41613 follow-up was recorded in months; its time-dependent AUCs were computed at 12, 24, and 36 months.

Cohort	Role	n	Events	C-Index	AUC 1y	AUC 2y	AUC 3y
TCGA-HNSC	Training (feature and model selection)	501	219	0.88	0.917	0.946	0.947
GSE42743	External model selection	74	42	0.68	0.758	0.760	0.682
E-MTAB-8588	Independent external test	83	53	0.62	0.568	0.635	0.662
GSE65858	Independent external test	270	94	0.58	0.634	0.608	0.587
GSE41613	Independent external test (AUCs at 12/24/36 months)	97	51	0.62	0.764	0.711	0.680

## Data Availability

All bulk RNA-seq, microarray, scRNA-seq, and spatial transcriptomics data used in this study are publicly available. Eleven GEO datasets (GSE29330, GSE30784, GSE3292, GSE42743, GSE6791, GSE7224, GSE9844, GSE65858, GSE41613, GSE234933, and GSE181300) were obtained from the NCBI Gene Expression Omnibus (https://www.ncbi.nlm.nih.gov/geo/, (accessed on 17 July 2026)). TCGA data were obtained from The Cancer Genome Atlas (https://www.cancer.gov/tcga, (accessed on 17 July 2026)). The E-MTAB-8588 dataset is available through ArrayExpress (https://www.ebi.ac.uk/arrayexpress/, (accessed on 17 July 2026)). Drug screening compound structures were sourced from the ZINC database (https://zinc.docking.org/). No new primary data were generated in this study. The analysis code and environment documentation for this study are available at https://github.com/dr-gu/HNSCC-SNI-public (accessed on 10 August 2026).

## Supplementary Materials

The following supporting information can be downloaded at https://www.mdpi.com/article/10.3390/biomedicines14092102/s1, Figure S1: Time-dependent receiver operating characteristic (ROC) curves for the SNI model in the external model-selection cohort GSE42743 and the three independent external test cohorts. Area-under-the-curve (AUC) values are shown at 1, 2, and 3 years for GSE42743, E-MTAB-8588, and GSE65858, and at 12, 24, and 36 months for GSE41613 (follow-up recorded in months); Figure S2: Kaplan–Meier overall survival curves of the high- versus low-SNI groups (median cut-off within each cohort) in the external model-selection cohort GSE42743 (n = 74) and the three independent external test cohorts (E-MTAB-8588, n = 83; GSE65858, n = 270; GSE41613, n = 97); Figure S3: Forest plot of per-unit-risk-score hazard ratios for overall survival in the external model-selection cohort GSE42743 and the three independent external test cohorts, with the cohort-stratified pooled estimate (n = 524); Figure S4: Spearman correlations between HSPH1 and ST6GALNAC1 expression and drug sensitivity (IC50) across the GDSC2 panel (805 cell lines, 198 drugs). The top 20 significant correlations (BH-adjusted *p* < 0.05) by |rho| are shown; red bars denote positive correlations (resistance) and blue bars negative correlations (sensitivity). Full results are provided in Table S4a; Figure S5: Spearman correlations between HSPH1 and ST6GALNAC1 expression and drug sensitivity (AUC) across the CTRP v2 panel (829 cell lines, 545 drugs). The top 20 significant correlations (BH-adjusted *p* < 0.05) by |rho| are shown; red bars denote positive correlations (resistance) and blue bars negative correlations (sensitivity). Full results are provided in Table S4b; Figure S6: Spearman correlations between HSPH1 and ST6GALNAC1 expression and drug sensitivity (AUC) across the DepMap PRISM panel (477 cell lines, 1448 drugs). The top 20 significant correlations (BH-adjusted *p* < 0.05) by |rho| are shown; red bars denote positive correlations (resistance) and blue bars negative correlations (sensitivity). Full results are provided in Table S4d; Figure S7: Spearman correlations between HSPH1 and ST6GALNAC1 expression and GDSC2-derived predicted drug-response scores (oncoPredict, with HSPH1 and ST6GALNAC1 removed from the feature space to avoid circularity) in TCGA-HNSC (n = 501). The top 20 significant correlations (BH-adjusted *p* < 0.05) by |rho| are shown; red bars denote positive correlations (predicted resistance) and blue bars negative correlations (predicted sensitivity). Full results are provided in Table S4c; Table S1: GeneCards sialylation compendium (1,204 genes) used for the DEG intersection; Table S2: Performance of the 117 machine learning algorithm combinations (nominally 101) benchmarked in the Mime1 framework; Table S3: SHAP threshold-sensitivity analysis: genes retained under top-K ∈ {3, 5, 10} and IQR cut-off ∈ {none, median, 75th percentile}; Table S4a: Spearman correlations between HSPH1 and ST6GALNAC1 expression and drug sensitivity (IC50) in the GDSC2 panel (805 cell lines, 198 drugs); Table S4b: Spearman correlations between HSPH1 and ST6GALNAC1 expression and drug sensitivity (AUC) in the CTRP v2 panel (829 cell lines, 545 drugs); Table S4c: Spearman correlations between HSPH1 and ST6GALNAC1 expression and GDSC2-derived predicted drug-response scores (higher scores indicate predicted resistance) in TCGA-HNSC (n = 501); Table S4d: Spearman correlations between HSPH1 and ST6GALNAC1 expression and drug sensitivity (AUC) in the DepMap PRISM panel (477 cell lines, 1448 drugs); Table S4a-HN: Spearman correlations between HSPH1 and ST6GALNAC1 expression and drug sensitivity (IC50) in the head-and-neck cancer subset of the GDSC2 panel (n = 29 cell lines).
