# Supplementary material for: From Glycan Biology to Drug Candidates: An Integrated Sialylation Niche Index and AI-Guided Therapeutic Framework for Head and Neck Squamous Cell Carcinoma
Source: Biomedicines. 2026 Sep 17;14(9):2102. doi: 10.3390/biomedicines14092102 (PMC13604810; doi:10.3390/biomedicines14092102)
Supplement: Supplementary file 1 [file biomedicines-14-02102-s001.zip › biomedicines-4523198-supplementary.pdf]

Supplementary Figures

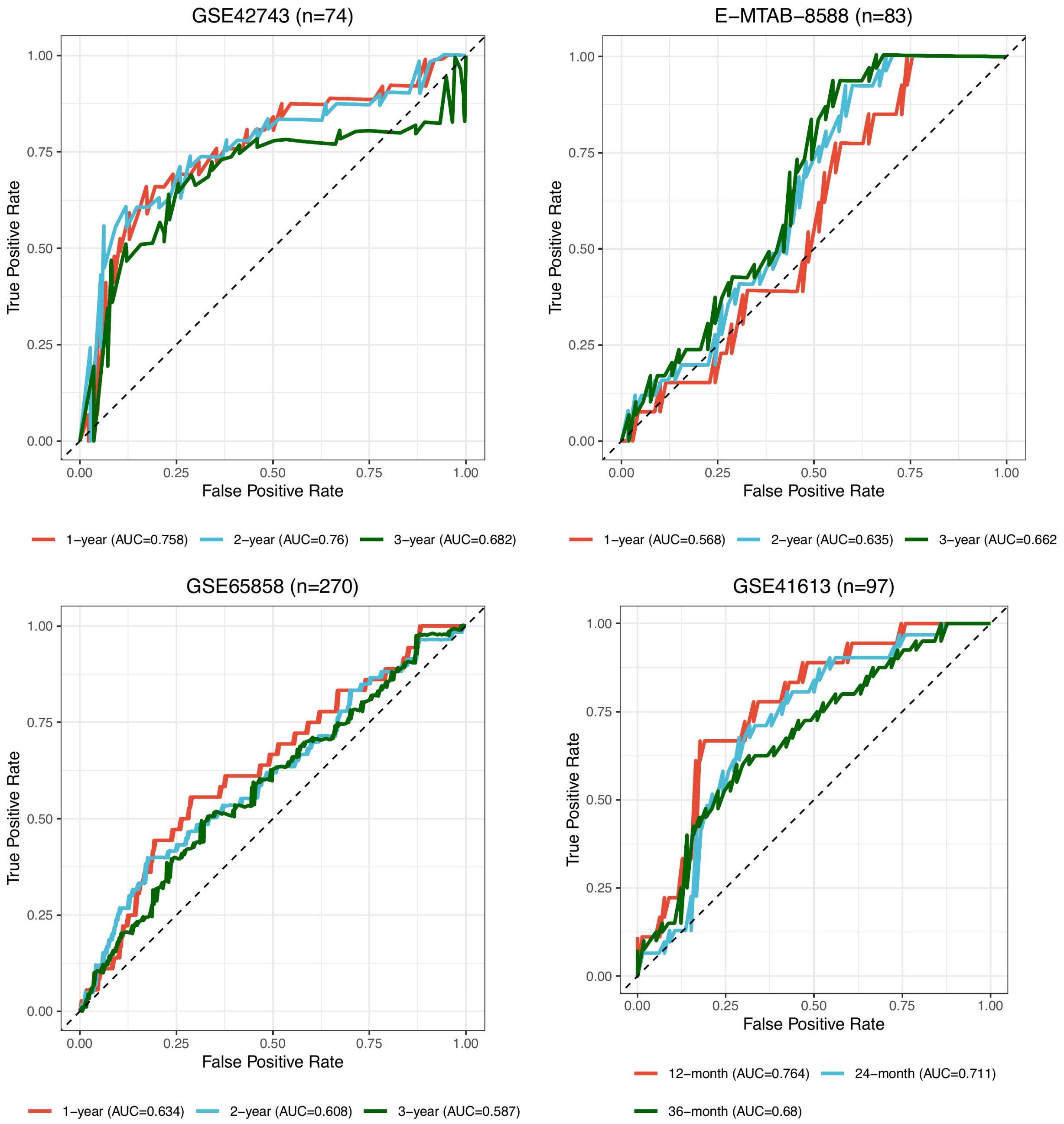

Figure S1. Time-dependent receiver operating characteristic (ROC) curves for the SNI model in the external model-selection cohort GSE42743 and the three independent external test cohorts. Area-under-the-curve (AUC) values are shown at 1, 2, and 3 years for GSE42743, E-MTAB-8588, and GSE65858, and at 12, 24, and 36 months for GSE41613 (follow-up recorded in months).

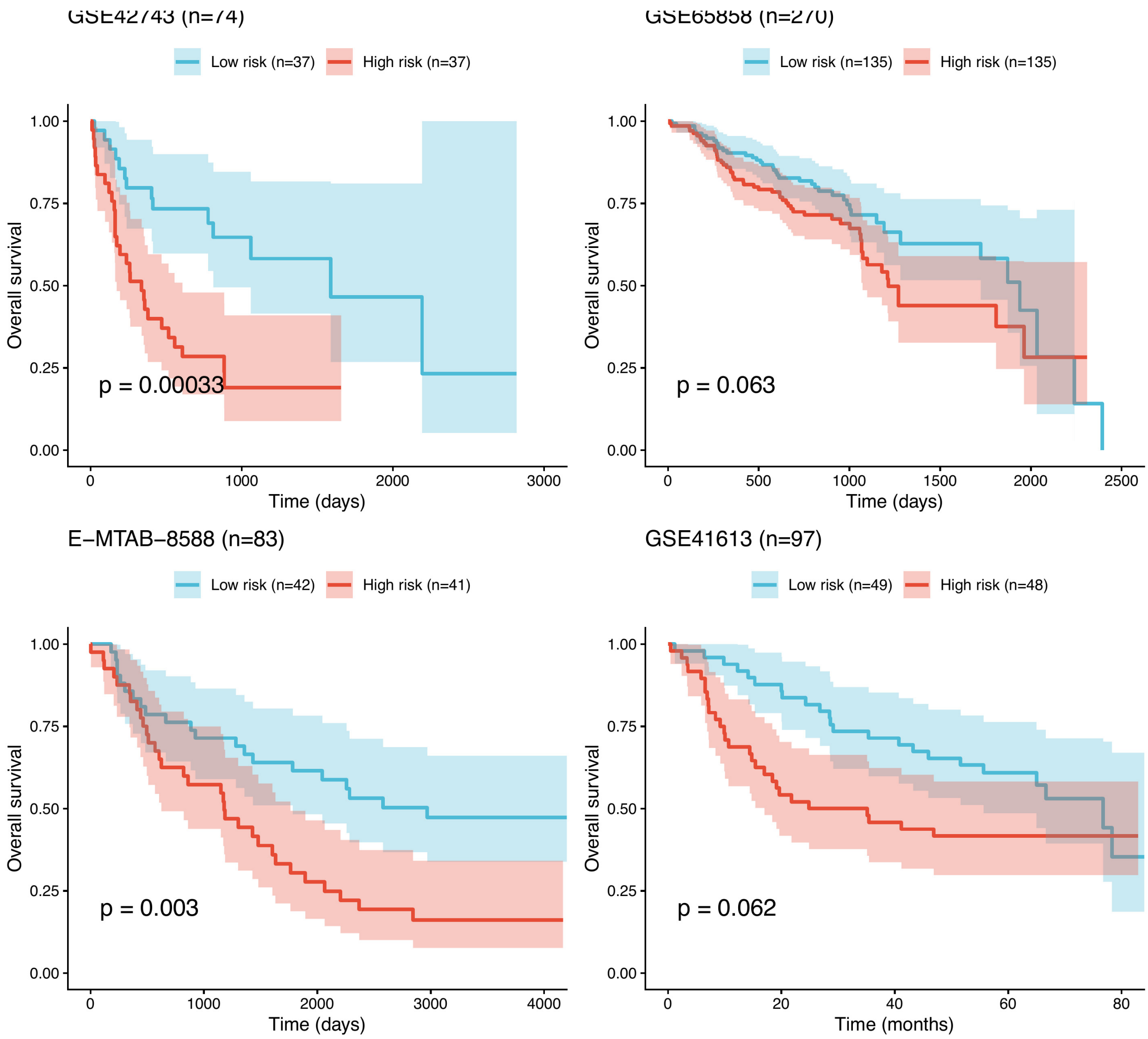

Figure S2. Kaplan–Meier overall survival curves of the high- versus low-SNI groups (median cut-off within each cohort) in the external model-selection cohort GSE42743 (n = 74) and the three independent external test cohorts (E-MTAB-8588, n = 83; GSE65858, n = 270; GSE41613, n = 97).

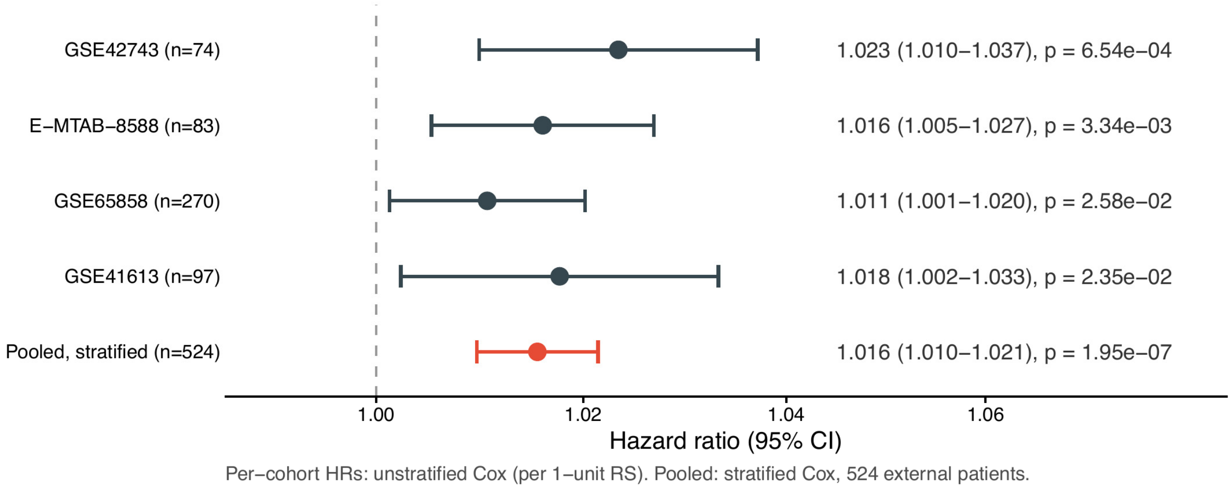

Figure S3. Forest plot of per-unit-risk-score hazard ratios for overall survival in the external model-selection cohort GSE42743 and the three independent external test cohorts, with the cohort-stratified pooled estimate (n = 524).

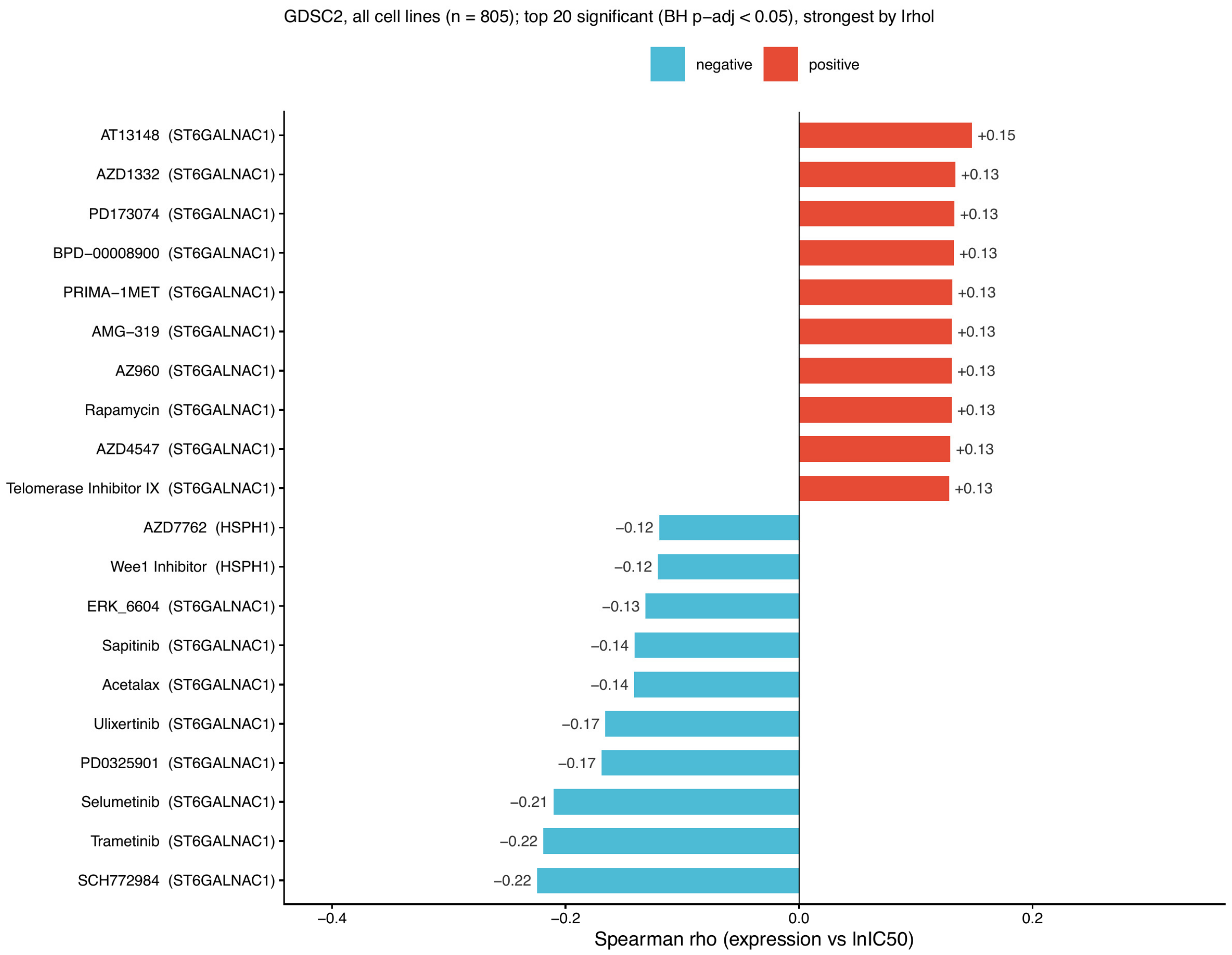

Figure S4. Spearman correlations between HSPH1 and ST6GALNAC1 expression and drug sensitivity (IC50) across the GDSC2 panel (805 cell lines, 198 drugs). The top 20 significant correlations (BH-adjusted $p < 0.05$) by |rho| are shown; red bars denote positive correlations (resistance) and blue bars negative correlations (sensitivity). Full results are provided in Table S4a.

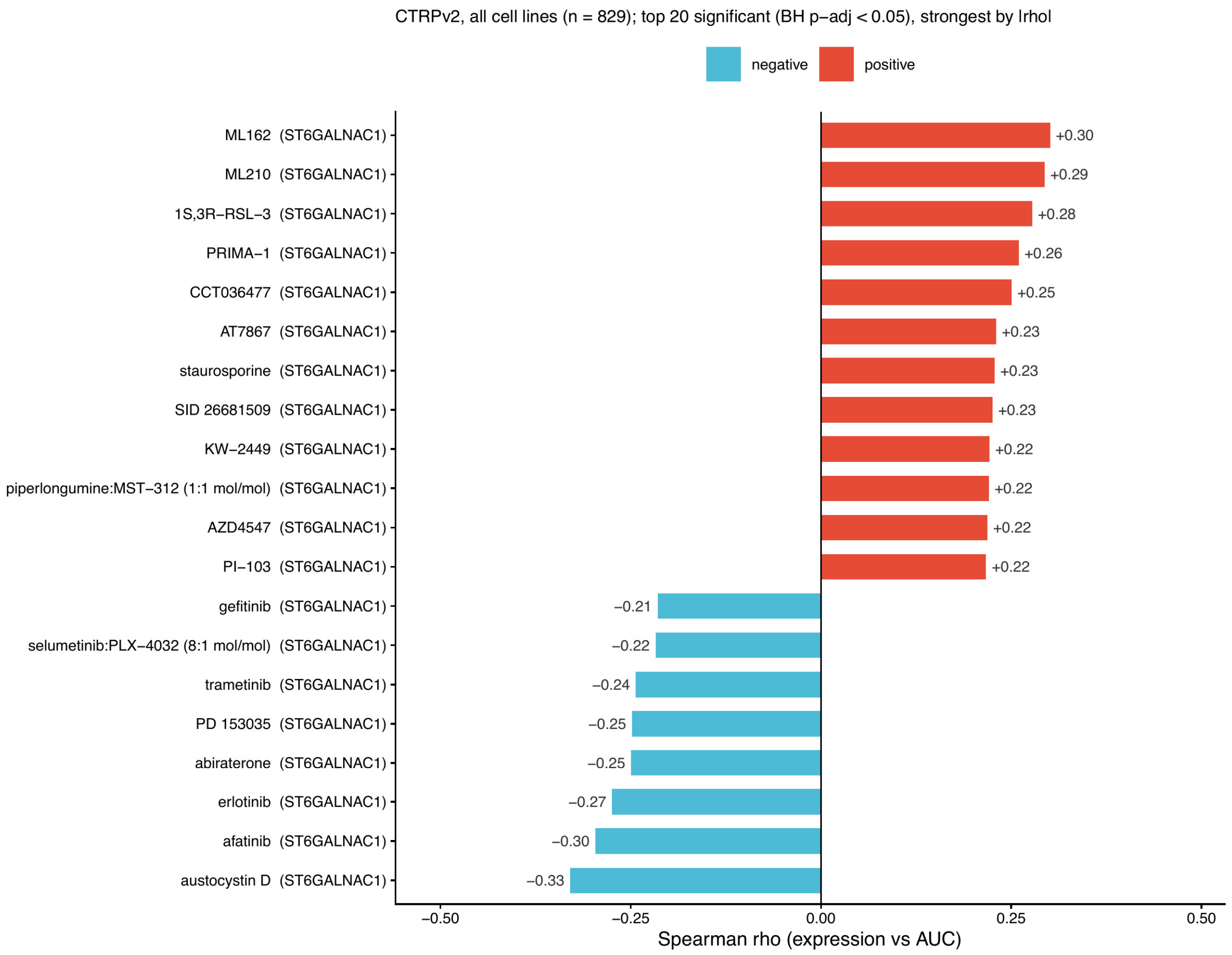

Figure S5. Spearman correlations between HSPH1 and ST6GALNAC1 expression and drug sensitivity (AUC) across the CTRP v2 panel (829 cell lines, 545 drugs). The top 20 significant correlations (BH-adjusted $p < 0.05$) by |rho| are shown; red bars denote positive correlations (resistance) and blue bars negative correlations (sensitivity). Full results are provided in Table S4b.

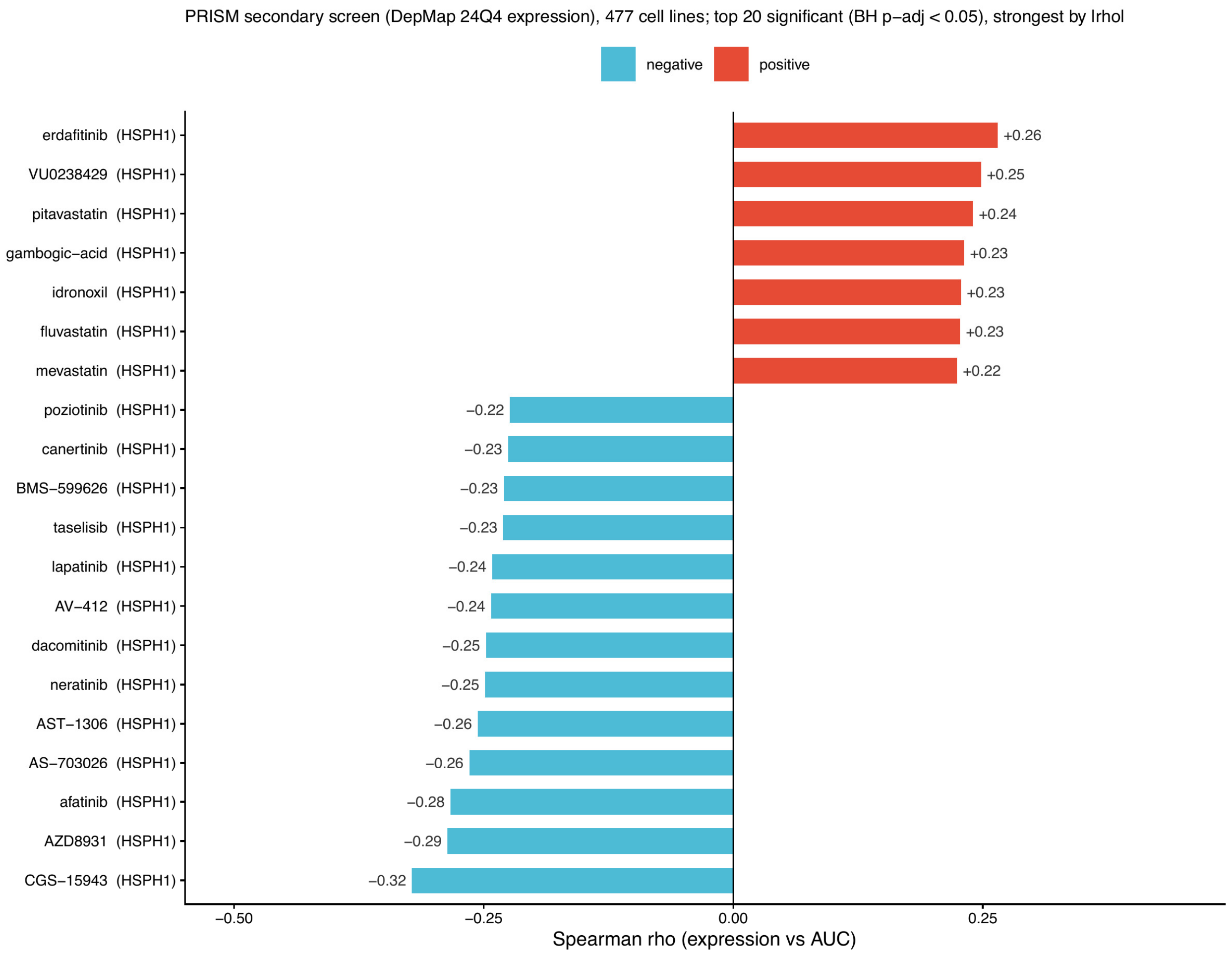

Figure S6. Spearman correlations between HSPH1 and ST6GALNAC1 expression and drug sensitivity (AUC) across the DepMap PRISM panel (477 cell lines, 1,448 drugs). The top 20 significant correlations (BH-adjusted p < 0.05) by |rho| are shown; red bars denote positive correlations (resistance) and blue bars negative correlations (sensitivity). Full results are provided in Table S4d.

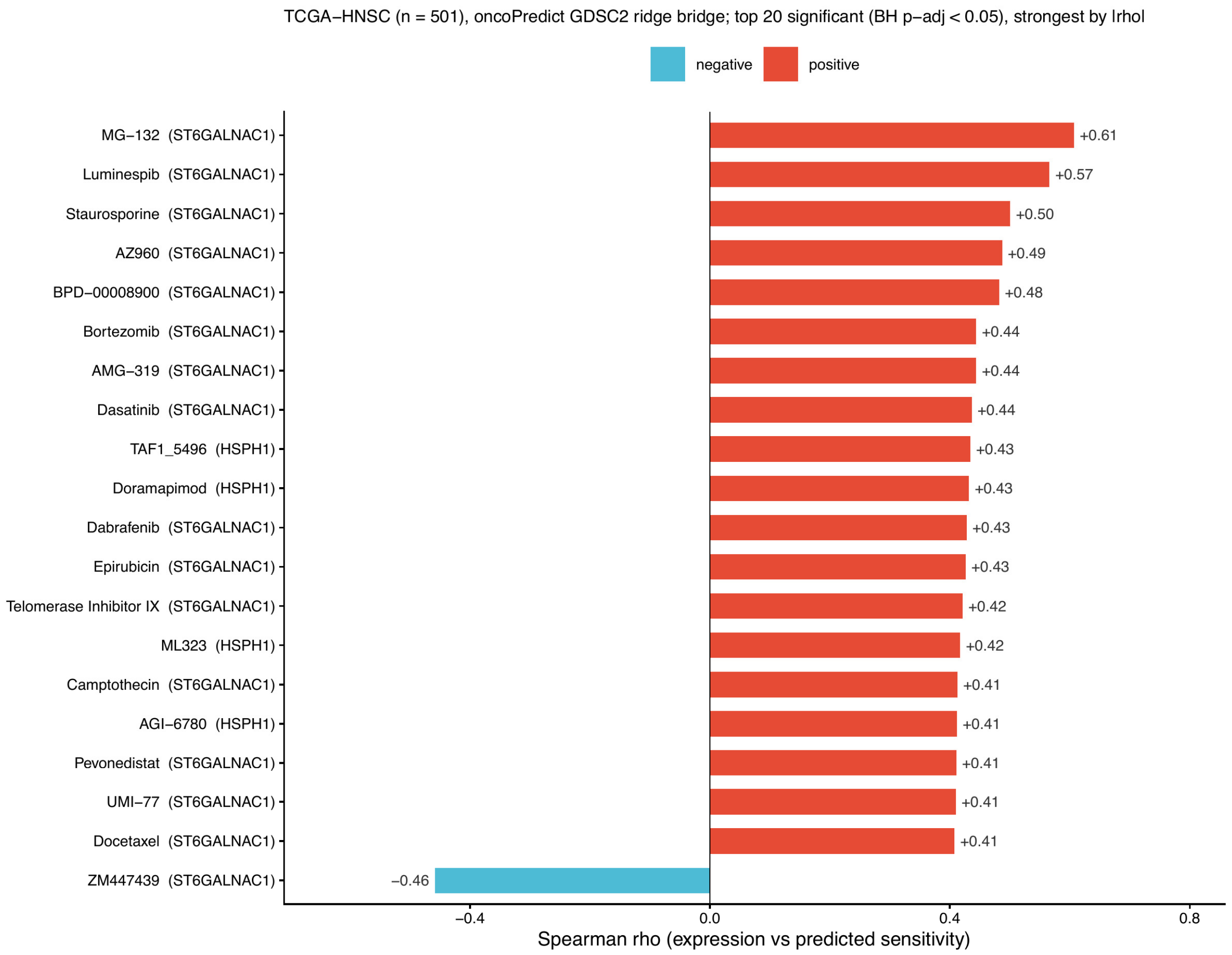

Figure S7. Spearman correlations between HSPH1 and ST6GALNAC1 expression and GDSC2-derived predicted drug-response scores (oncoPredict, with HSPH1 and ST6GALNAC1 removed from the feature space to avoid circularity) in TCGA-HNSC (n = 501). The top 20 significant correlations (BH-adjusted $p < 0.05$) by |rho| are shown; red bars denote positive correlations (predicted resistance) and blue bars negative correlations (predicted sensitivity). Full results are provided in Table S4c.
